# CaMKII induces permeability transition through Drp1 phosphorylation during chronic β-AR stimulation

**DOI:** 10.1038/ncomms13189

**Published:** 2016-10-14

**Authors:** Shangcheng Xu, Pei Wang, Huiliang Zhang, Guohua Gong, Nicolas Gutierrez Cortes, Weizhong Zhu, Yisang Yoon, Rong Tian, Wang Wang

**Affiliations:** 1 Mitochondria and Metabolism Center, Department of Anesthesiology and Pain Medicine, University of Washington, Seattle, Washington 98109, USA; 2 Department of Occupational Health, Third Military Medical University, Chongqing 400038, China; 3 Nantong University School of Pharmacy, Nantong, Jiangsu 226001, China; 4 Department of Physiology, Georgia Regents University, Augusta, Georgia 30912, USA

## Abstract

Mitochondrial permeability transition pore (mPTP) is involved in cardiac dysfunction during chronic β-adrenergic receptor (β-AR) stimulation. The mechanism by which chronic β-AR stimulation leads to mPTP openings is elusive. Here, we show that chronic administration of isoproterenol (ISO) persistently increases the frequency of mPTP openings followed by mitochondrial damage and cardiac dysfunction. Mechanistically, this effect is mediated by phosphorylation of mitochondrial fission protein, dynamin-related protein 1 (Drp1), by Ca^2+^/calmodulin-dependent kinase II (CaMKII) at a serine 616 (S616) site. Mutating this phosphorylation site or inhibiting Drp1 activity blocks CaMKII- or ISO-induced mPTP opening and myocyte death *in vitro* and rescues heart hypertrophy *in vivo*. In human failing hearts, Drp1 phosphorylation at S616 is increased. These results uncover a pathway downstream of chronic β-AR stimulation that links CaMKII, Drp1 and mPTP to bridge cytosolic stress signal with mitochondrial dysfunction in the heart.

## Abstract (web summary)

β-adrenergic receptor signaling induces mitochondrial permeability transition pore (mPTP) opening. Here, Xu *et al*. show that this effect is mediated by phosphorylation of mitochondrial fission protein Drp1 by CamKII, which increases the frequency of transient mPTP opening.

## Graphical abstract

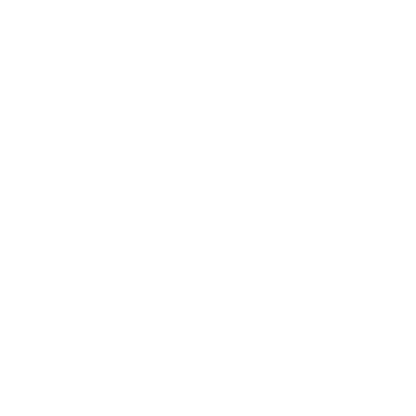

Activation of β-adrenergic receptors (β-AR) plays an essential role in stimulating cardiac output during fight-or-flight response^1^ and a deleterious role in heart failure^2^. Acute stimulation of β-AR augments cardiac contraction by increasing intracellular Ca^2+^ handling^3^, whereas chronic β-AR stimulation exhibits detrimental outcomes, such as myocardial hypertrophy and heart failure, mainly through activating Ca^2+^/calmodulin-dependent kinase II (CaMKII)^4^,^5^. Openings of mitochondrial permeability transition pore (mPTP) were involved in cardiotoxicity induced by chronic β-AR stimulation and preventing mPTP openings could attenuate mitochondrial stress and mitochondrial-dependent apoptosis^6^. Consistently, mitochondrial-targeted CaMKII inhibition efficiently prevented isoproterenol (ISO)-induced myocardial injury^7^. It is currently not known whether CaMKII plays a causal role in mPTP openings during chronic β-AR stimulation. Uncovering the molecular mechanism or signalling pathway that mediates chronic β-AR stimulation-induced mPTP openings bears tremendous clinical significance, due to the fact that high catecholamine levels and chronic β-AR activation are hallmarks of human heart failure.

As a crucial gatekeeper, transient or subconductance openings of mPTP occur under physiological conditions and critically maintain mitochondrial homeostasis^8^,^9^. On the other hand, massive or prolonged mPTP openings, which can be triggered by Ca^2+^ overload, oxidative stress and mitochondrial fission/fusion, uncouple oxidative phosphorylation, induce mitochondrial swelling, promote cytochrome c release, and contribute to the pathology of heart disease such as ischaemia reperfusion injury^10^,^11^. Tracking mPTP activity in intact cells or *in vivo* poses a major technical challenge. Recently, we and others have developed the optical recording of short-term and reversible mPTP events by imaging single mitochondrial flashes^12^. Despite debates over the nature of flashes^13^, consensus has been reached that these events are triggered by mPTP and fuelled by mitochondrial electron transport chain in the heart^12^,^14^,^15^,^16^. This breakthrough discovery provides a tool for evaluating mPTP in the healthy and diseased heart.

By utilizing this state-of-the-art technology, we found that chronic β-AR stimulation induces mPTP through activating CaMKII and subsequent phosphorylation of dynamin-related protein 1 (Drp1) at S616. Inhibiting CaMKII activity, Drp1 phosphorylation or Drp1 activity suppressed mPTP openings, ameliorated mitochondrial dysfunction and myocyte death *in vitro*, and rescued heart dysfunction *in vivo*. Taken together, we identify a new mechanism by which chronic β-AR stimulation induces mitochondrial dysfunction through post-translational modification of a fission protein in the heart.

## Results

### Chronic β-AR stimulation augmented mPTP opening

**Figure 1:**
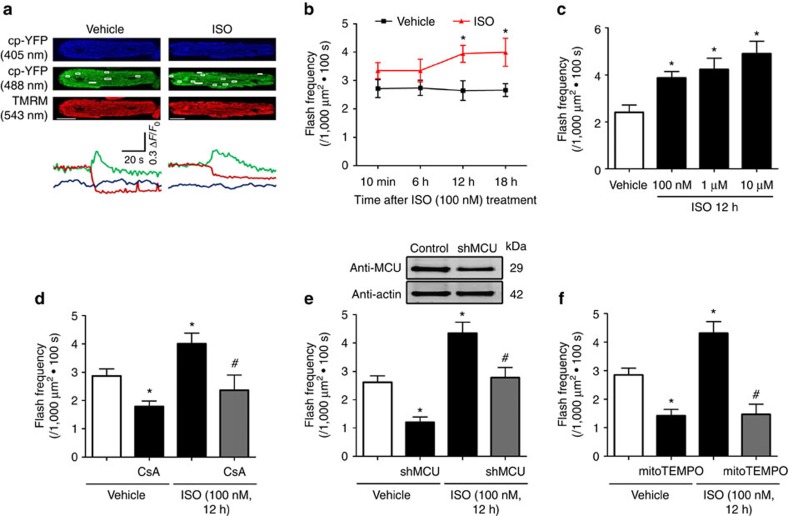
Chronic ISO stimulation persistently elevated mPTP openings. (**a**) Representative images and traces of the onset of mitochondrial flashes (white boxes) colocalized with Δ*ψ*_m_ indicator TMRM in adult rat cardiomyocytes treated with or without ISO (100 nM for 12 h). Scale bars, 10 μm. (**b**) Time-course assay showing the effects of ISO treatment on mitochondrial flash frequency in adult cardiomyocytes. In Vehicle groups, *N*=27, 40, 31, and 58 cells from 3 to 6 rats in the time points of 10 min, 6, 12 and 18 h. In ISO groups, *N*=43, 26, 50 and 23 in the time points of 10 min, 6, 12 and 18 h. *P<0.05 versus Vehicle at the same time point. (**c**) ISO treatment increased flash frequency in a dose-dependent manner. *N*=31, 37, 18 and 12 cells from 3 to 6 rats in the groups of Vehicle, 100 nM, 1 μM and 10 μM ISO. *P<0.05 versus Vehicle group. (**d**) Cyclophilin D inhibitor, CsA, (1 μM, 30 min) abolished the increase of flash frequency induced by ISO (100 nM, 12 h). *N*=26, 13, 19 and 22 cells from 3 to 6 rats in the groups of Vehicle, CsA, ISO and CsA+ISO, respectively. *P<0.05 versus Vehicle group, ^#^*P*<0.05 versus ISO group. (**e**) Knocking down mitochondrial Ca^2+^ uniporter (MCU) by short hairpin RNA (shMCU) blocked ISO-induced flashes. *N*=39, 14, 30 and 17 cells from 3 to 7 rats in the groups of Vehicle, shMCU, ISO and shMCU+ISO, respectively. *P<0.05 versus Vehicle group, ^#^*P*<0.05 versus ISO group. (**f**) Pretreatment with mitochondrial antioxidant, mitoTEMPO (1 μM, 1 h) attenuated ISO-induced flashes. *N*=37, 11, 37 and 11 cells from 3 to 6 rats in the groups of Vehicle, mitoTEMPO, ISO and mitoTEMPO+ISO, respectively.*P<0.05 versus Vehicle group, ^#^*P*<0.05 versus ISO group. Data in **b**–**f** are mean±s.e.m. The data were analysed using Student's *t*-test in **b** and One-way ANOVA followed by Turkey post-test in **c**–**f**.

To evaluate the effect of chronic β-AR stimulation on mPTP, we incubated adult cardiomyocytes with physiological levels of ISO (100 nM) for up to 18 h. ISO treatment augmented flash frequency starting at 12 h (1.5 fold over control) and persisting to 18 h (Fig. 1a,b). Unitary features of flash including amplitude, rising time and decay kinetics were not significantly changed (Supplementary Fig. 1a–c). The ISO-induced flashes were also coincided with loss of mitochondrial membrane potential (Δ*ψ*_m_, Fig. 1a) consistent with previous reports^12^. ISO also dose-dependently increased flash frequency (Fig. 1c). ISO-induced increase in mitochondrial flash frequency was largely blocked by pre-incubation of mPTP inhibitor, cyclosporine A (CsA, 1 μM), knocking down mitochondrial Ca^2+^ uniporter (MCU), or a mitochondria-targeted superoxide scavenger, mitoTEMPO (1 μM) suggesting the mPTP dependence of flash (Fig. 1d–f and Supplementary Fig. 1d). Taken together, chronic β-AR stimulation persistently induced mitochondrial flash-coupled mPTP in a frequency-dependent mode.

### Chronic β-AR stimulation-induced myocyte dysfunction

Chronic ISO treatment increased the sensitivity of mitochondria to oxidative stress as shown by a shorter time to laser-induced Δ*ψ*_m_ loss and decreased resting Δ*ψ*_m_ suggesting mitochondrial damage (Supplementary Fig. 2a–d). Meanwhile, ISO also induced myocyte dysfunction as evidenced by increased oxidative stress at 24 h, decreased Ca^2+^ transient amplitude, compromised cardiac contraction, and increased both necrosis and apoptosis at 48 h (Supplementary Fig. 2e–i). Thus, chronic β-AR stimulation elicited sequential events in mitochondrial and myocyte dysfunction in adult cardiomyocytes (Supplementary Fig. 2j). Despite the significant impact on mitochondrial and myocyte function, mitochondrial biogenesis and autophagy were not significantly altered by ISO stimulation *in vitro* (100 nM, 12–48 h) or *in vivo* (15 mg kg^−1^, 2 weeks) (Supplementary Fig. 3).

**Figure 2:**
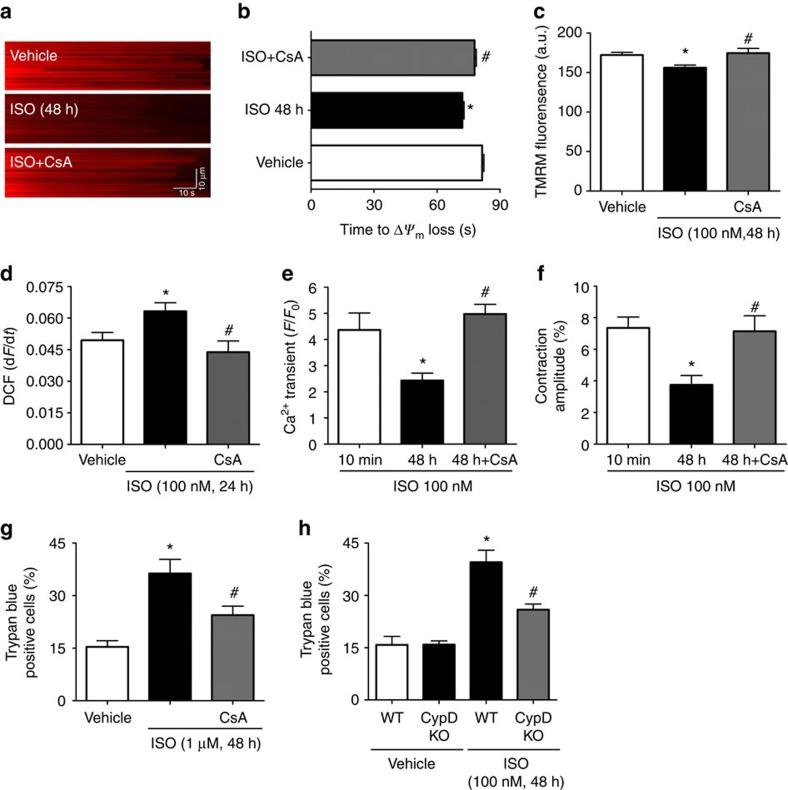
Blocking ISO-induced mPTP openings prevented mitochondrial and myocyte dysfunction during chronic β-AR stimulation. (**a**) Representative linescan confocal images showing the laser-induced permanent loss of Δ*ψ*_m_ in individual mitochondrion. CsA (1 μM) was added 12 h after ISO treatment, when the ISO-induced flash frequency reaches peak. (**b**) Quantification of the time from the start of scan to the sudden loss of Δ*ψ*_m_. The shorter the time, the more sensitive the mitochondria to the laser. *N*=312, 383 and 274 mitochondria from 23 to 30 cells and three rats in the groups of Vehicle, ISO and ISO+CsA, respectively. (**c**) The intensity of TMRM fluorescence at the beginning of each scan was used to evaluate the basal Δ*ψ*_m_ in individual mitochondrion. (**d**) CsA reversed ISO-induced cellular oxidative stress reflected by the increased rate of DCF-DA fluorescence over 30 s (1 frame per second) (d*F*/d*t*). *N*=46, 53 and 22 cells from four rats in the groups of Vehicle, ISO and ISO+CsA, respectively. (**e**,**f**) CsA attenuated the reduction of Ca^2+^ transients (**e**) and cell contraction amplitude (**f**) after 48 h ISO treatment. *N*=21, 22 and 24 cells from three rats in the groups of Vehicle, ISO and ISO+CsA, respectively. (**g**) CsA rescued myocyte death after 48 h of ISO (1 μM) treatment measured by Trypan blue assay. *N*=903, 643 and 604 cells from four rats in the groups of Vehicle, ISO and ISO+CsA, respectively. (**h**) Knockout of CypD ameliorated cell death in cultured adult mouse cardiomycytes treated with ISO (100 nM, 48 h). *N*=637, 651, 565 and 693 cells from three mice in the groups of Vehicle, CypD KO, ISO and CypD KO+ISO, respectively. Data in **b**–**h** are mean±s.e.m. *P<0.05 versus Control group, ^#^*P*<0.05 versus ISO group. The data were analysed using One-way ANOVA followed by Turkey post test in **b**–**h**.

To test whether mPTP underlies ISO-induced mitochondrial and myocyte dysfunction, we blocked mPTP opening by CsA, at 12 h after ISO treatment when increased flash-coupled mPTP was detected, and found that CsA prevented laser-induced Δ*ψ*_m_ loss suggesting it decreased mPTP sensitivity, maintained Δ*ψ*_m_, and decreased cellular oxidative stress at 24–48 h (Fig. 2a–d). Moreover, CsA added at 12 h after ISO stimulation also enhanced Ca^2+^ transient amplitude and contractility and rescued myocyte death at 48 h (Fig. 2e–g). To obtain molecular evidence on the mPTP dependence of ISO-induced mitochondrial and myocyte dysfunction, we treated adult cardiomyocytes from cyclophilin D knockout (CypD KO) mice with ISO. CypD KO myocytes were resistant to chronic ISO-induced cell death (Fig. 2h) consistent with previous report^17^. Taken together, increased mPTP openings are responsible for mitochondrial and cardiac dysfunction in chronic β-AR stimulation.

### Chronic β-AR stimulation triggered mPTP through CaMKII

**Figure 3:**
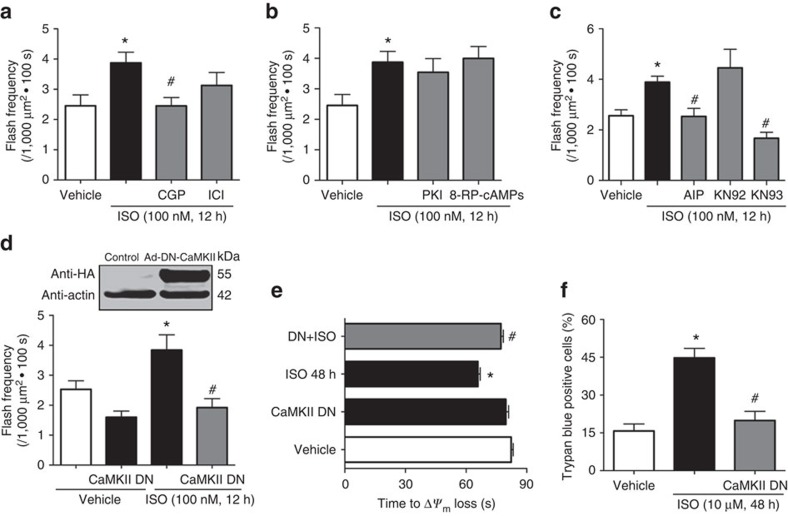
ISO induced mPTP openings through β1-AR and CaMKII pathway. (**a**) Pretreatment with β1-AR specific antagonist (CGP, 0.5 μM), but not β2-AR specific antagonist (ICI, 0.5 μM), blocked the ISO-induced flashes. *N*=38, 48, 36 and 31 cells from 3 to 5 rats in the groups of Vehicle, ISO, CGP+ISO and ICI+ISO, respectively. (**b**) Either the peptide inhibitor for PKA (PKI, 10 μM) or a cell-permeable inactive cAMP analogue (8-RP-cAMPs, 100 μM) failed to prevent the increase of flash after ISO treatment. *N*=25, 34, 20 and 19 cells from three rats in the groups of Vehicle, ISO, PKI+ISO and 8-RP+ISO, respectively. (**c**) CaMKII blockers, AIP (10 μM) and KN-93 (0.5 μM), inhibited ISO-induced flashes. KN-92, the inactive analogue of KN-93, was used as control. *N*=44, 55, 29, 30 and 31 cells from 3 to 5 rats in the groups of Vehicle, ISO, AIP+ISO, KN93+ISO and KN92+ISO, respectively. All the reagents in **a**–**c** were added to the myocytes 30 min before ISO treatment. (**d**) Inhibition of CaMKII activity by adenovirus-mediated overexpression of a dominant negative CaMKII (CaMKII DN) significantly attenuated mPTP openings 12 h after ISO treatment. *N*=20, 22, 18 and 20 cells from three rats in the groups of Vehicle, CaMKII DN, ISO and CaMKII DN+ISO, respectively. (**e**) CaMKII DN overexpression prolonged the time of laser-induced permanent loss of Δ*ψ*_m_ after ISO treatment (100 nM, 48 h). *N*=208, 179, 260 and 165 mitochondria from 15 to 25 cells and three rats in the groups of Vehicle, CaMKII DN, ISO and CaMKII DN+ISO, respectively. (**f**) CaMKII DN overexpression prevented ISO-induced cell death (1 μM, 48 h). *N*=474, 577 and 374 cells from four rats in the group of Vehicle, ISO and CaMKII DN+ISO, respectively. Data in **a**–**f** are mean±s.e.m. *P<0.05 versus Control group, ^#^*P*<0.05 versus ISO group. The data were analysed using One-way ANOVA followed by Turkey post test in **a**–**f**.

Next, we explored the signalling pathways mediating chronic β-AR stimulation-induced mPTP openings. First, β1-AR blocker (CGP 20712A, 0.5 μM), but not β2-AR blocker (ICI 118,551, 0.5 μM), attenuated ISO-induced flash activity (Fig. 3a), suggesting chronic ISO stimulation mainly targets β1-AR (refs 2, 5). Second, previous work suggests that protein kinase A (PKA) pathway underlies the acute responses of β1-AR activation, while CaMKII pathway is activated 3–6 h later and persistently for up to 24 h (ref. 18). Indeed, neither PKA inhibitor peptide (PKI, 10 μM) nor an inactive cAMP analogue (Rp-PIP-cAMP, 100 μM), had any effect on chronic ISO-induced flash activity (Fig. 3b). In contrast, pretreatment with CaMKII specific inhibitory peptide (autocamtide 2-related inhibitory peptide (AIP), 10 μM), abolished ISO-induced flash activity. Similar effects were observed with another specific CaMKII inhibitor, KN93 (0.5 μM), but not its inactive analogue, KN92 (2 μM, Fig. 3c). In parallel, we detected increased phospholamban phosphorylation at threonine 17 site (PLN (ref. 17)), a CaMKII specific site, during chronic ISO treatment, which was prevented by KN93 (Supplementary Fig. 4a,b). Therefore, ISO persistently activates mPTP through CaMKII but not PKA pathway.

To further explore the molecular mechanism on how CaMKII induces mPTP, we overexpressed the dominant-negative CaMKII (CaMKII DN)^19^, which prevented ISO-induced flash activity (Fig. 3d). The effectiveness of CaMKII DN in blocking endogenous CaMKII activity is confirmed by decreased PLN (ref. 17) phosphorylation (Supplementary Fig. 4c). Taken together, ISO-induced mPTP is through CaMKII pathway. In addition, CaMKII DN overexpression prevented ISO-induced mitochondrial dysfunction, as indicated by a longer time of laser-induced Δ*ψ*_m_ loss, and rescued myocyte death (Fig. 3e,f). These results suggest that CaMKII mediates ISO-induced mPTP and cardiotoxicity.

**Figure 4:**
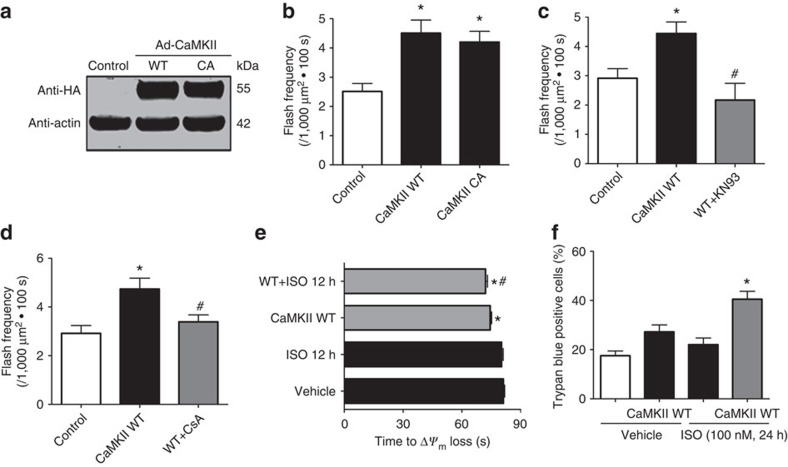
Activating CaMKII pathway stimulated mPTP opening and mitochondrial stress in adult cardiomyocytes. (**a**) Representative images of Western blot using the anti-HA antibody confirmed the expression of HA-tagged wild-type CaMKII (CaMKII WT) and a constitutively active CaMKII (CaMKII CA). (**b**) Overexpression of CaMKII WT or CaMKII CA significantly increased flash frequency in adult cardiomyocytes. *N*=26, 28 and 26 cells from four rats in the groups of control, CaMKII WT and CaMKII CA, respectively. *P<0.05 versus control group. (**c**) Inhibition of CaMKII activity by applying KN93 (0.5 μM) attenuated the increased flash frequency by CaMKII WT overexpression. *N*=7, 9 and 9 cells from three rats in the groups of Control, CaMKII WT and WT+KN93, respectively. *P<0.05 versus control group, ^#^*P*<0.05 versus CaMKII WT group. (**d**) CsA (1 μM) inhibited the increased mPTP openings by CaMKII WT overexpression. *N*=14, 27 and 22 cells from three rats in the groups of control, CaMKII WT and WT+KN93, respectively. *P<0.05 versus Control group, ^#^*P*<0.05 versus CaMKII WT group. (**e**) CaMKII WT overexpression promoted laser-induced permanent loss of Δ*ψ*_m_ in adult cardiomyocytes and showed additive effect with ISO treatment (100 nM, 12 h). *N*=536, 366, 305 and 242 mitochondria from 21 to 48 cells and four rats in the groups of control, ISO, CaMKII WT and WT+ISO, respectively. *P<0.05 versus Vehicle group, ^#^*P*<0.05 versus CaMKII WT group. (**f**) CaMKII WT overexpression exaggerated cell death after ISO treatment (100 nM, 24 h). *N*=665, 577, 1,216 and 567 cell from five rats in the groups of Vehicle, CaMKII WT, ISO and WT+ISO, respectively. *P<0.05 versus ISO 24 h group. Data in **b**–**f** are mean±s.e.m. The data were analysed using One-way ANOVA followed by Turkey post test in **b**–**f**.

Next, we manipulated CaMKII activity through a gain of function approach. Overexpression of wild-type CaMKII (CaMKII WT) or a constitutively active CaMKII (CaMKII CA) increased flash activity (Fig. 4a,b) and PLN (ref. 17) phosphorylation (Supplementary Fig. 4c). CaMKII specific inhibitor (KN93, 0.5 μM) or mPTP inhibitor (CsA, 1 μM) blocked the effect of CaMKII WT (Fig. 4c,d). Overexpression of CaMKII also potentiated the effect of ISO on laser-induced Δ*ψ*_m_ loss and cardiomyocyte death as indicated by the earlier onset of these events (Fig. 4e,f). Thus increased CaMKII activity is sufficient to induce mPTP and myocyte dysfunction.

**Figure 5:**
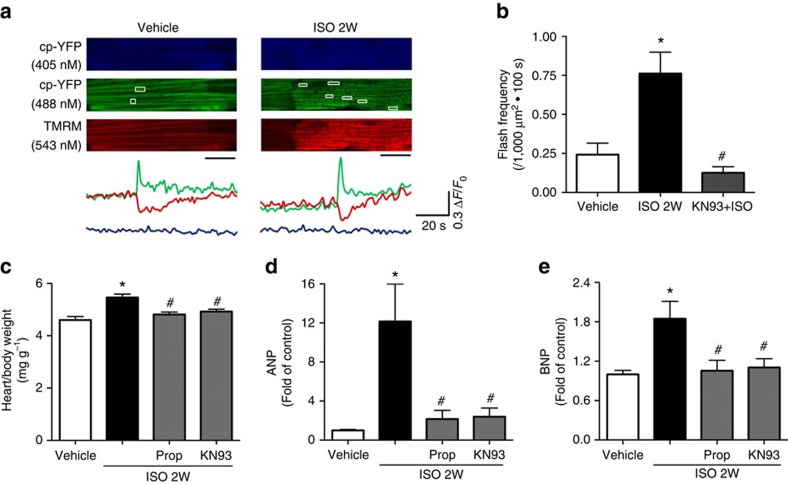
Chronic ISO infusion *in vivo* increased mPTP openings in the intact heart of mt-cpYFP transgenic mice and induced cardiac hypertrophy. (**a**) Representative images and traces showing the onset of mitochondrial flashes (white boxes) accompanied by loss of Δ*ψ*_m_ (TMRM signal) in intact and Langendorff perfused heart. Scale bar, 10 μm. The mice were under ISO infusion (15 mg kg^−1^ d^−1^ for 2 weeks, ISO 2W) by a mini-osmotic pump implanted subcutaneously. (**b**) Summarized data showing the increase in flash frequency by ISO infusion and its blockade by KN93 (10 μM kg^−1^ administered together with ISO). *N*=33, 27 and 25 serial scanning images from 4 to 6 mice in the groups of Vehicle, ISO and KN93+ISO, respectively. (**c**–**e**) ISO-induced cardiac hypertrophy was measured by heart weight to body weight ratio (**c**, *N*=5) and quantitative real-time PCR for ANP (**d**, *N*=4) and BNP (**e**, *N*=6). KN93 or β-blocker (Prop, 10 mg kg^−1^) were administered together with ISO. Data in **b**–**e** are mean±s.e.m. *P<0.05 versus Vehicle group, ^#^*P*<0.05 versus ISO group. The data were analysed using One-way ANOVA followed by Turkey post test in **b**–**e**.

Finally, infusing mt-cpYFP transgenic mice with ISO (15 mg kg^−1^ for 14 days), a well-established model for heart hypertrophy, resulted in significantly increased flash frequency detected in the perfused intact heart (Fig. 5a). Importantly, administration of KN93 (10 μM kg^−1^) together with ISO prevented this increase (Fig. 5b). Moreover, propranolol or KN93 reversed the increased heart/body weight ratio and hypertrophic markers (ANP and BNP) (Fig. 5c–e). Thus, the ISO–CaMKII–mPTP pathway exists in *in vivo* conditions.

Since CaMKII can be activated by oxidation^20^,^21^, we tested whether oxidative stress underlies ISO-induced CaMKII activation. Through monitoring mitochondrial reactive oxygen species (ROS) production by MitoSOX red or a mitochondria-targeted genetic H_2_O_2_ indicator, mtHyper^22^,^23^, we found no increase in mitochondrial ROS 12–24 h after ISO treatment (Supplementary Fig. 5). CaMKII is activated and flash-coupled mPTP openings are increased at 6–12 h after ISO treatment. Thus, it seems that oxidative stress cannot be account for the initial activation of this pathway. We do detected increased cytosolic ROS 24 h after ISO treatment (Fig. 2d), which is consistent with previous reports^6^,^24^. Therefore, it is likely that oxidative stress is downstream of mPTP and may act in a positive feedback manner to support sustained CaMKII activation and mPTP openings at the later stage^21^.

### CaMKII induced mPTP via Drp1 S616 phosphorylation

**Figure 6:**
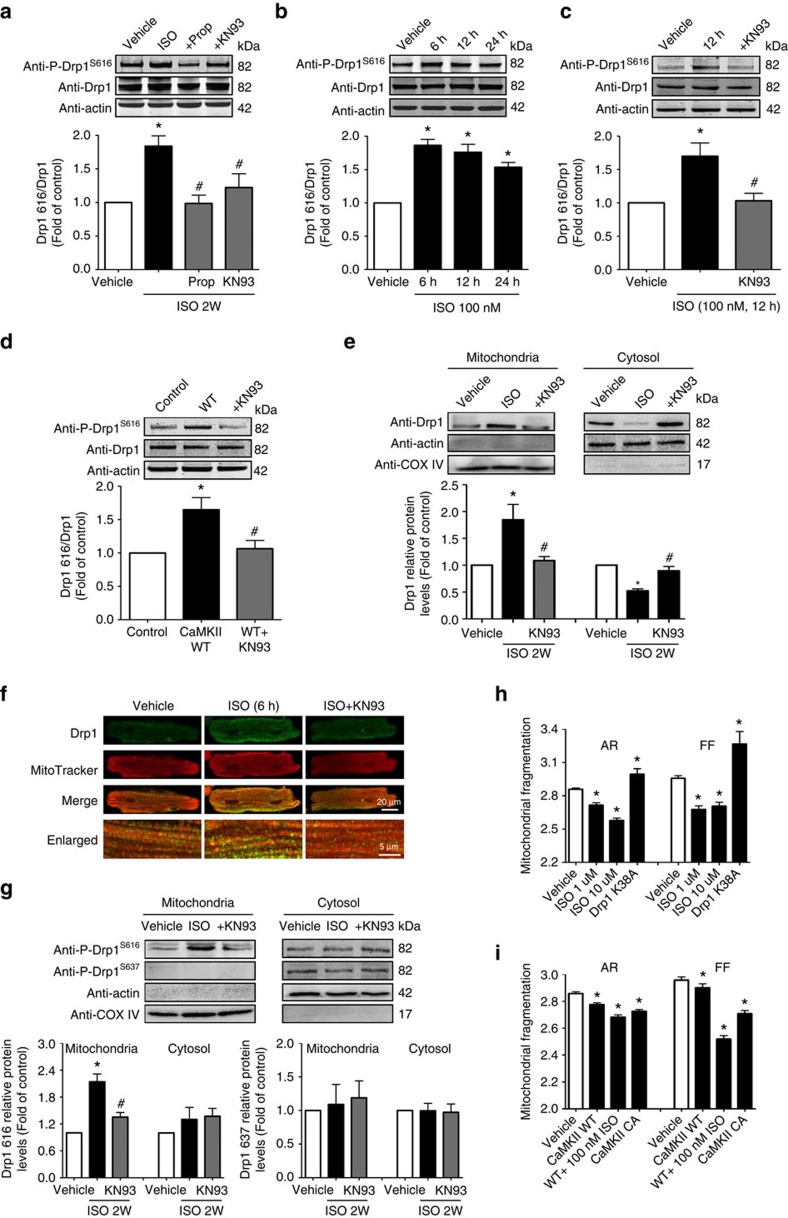
Chronic ISO administration promoted Drp1 S616 phosphorylation and its mitochondrial translocation through CaMKII pathway. (**a**) Increased Drp1 phosphorylation at S616 site (Drp1^S616^) in the heart after ISO infusion through CaMKII-dependent pathway. *N*=5, 6, 4 and 4 mice in the groups of Vehicle, ISO, Prop+ISO and KN93+ISO, respectively. (**b**) ISO administration induced Drp1^S616^ phosphorylation in cultured adult rat cardiomyocytes. *N*=6. (**c**) CaMKII blocker, KN-93 (0.5 μM), prevented Drp1^S616^ phosphorylation by ISO (100 nM for 12 h). *N*=4. (**d**) CaMKII WT overexpression induced Drp1^S616^ phosphorylation in adult cardiomycytes. *N*=4. (**e**) Representative immunoblots and quantification of Drp1 proteins in mitochondrial and cytosolic fractions of the heart after ISO infusion. COX IV and β-actin were used as mitochondrial and cytosolic markers, respectively. *N*=5, 6 and 4 mice in the groups of Vehicle, ISO and KN93+ISO, respectively. (**f**) Immunofluorescent analysis showing increased ‘punctate' Drp1 staining colocalized with mitochondria. Images are representative of 30 cells from three rats in each group. (**g**) Representative immunoblots and quantification of Drp1^S616^ or Drp1^S637^ phosphorylation in the mitochondrial or cytosolic fractions of the heart after 2-weeks of ISO infusion. *N*=4. (**h**) ISO treatment (1 μM or 10 μM for 24 h) induced mitochondrial fragmentation in H9C2 cardiac myoblasts. A dominant negative Drp1 mutation (Drp1 K38A) was used as positive control. Form factor (FF; the reciprocal of circularity value) and aspect ratio (AR; major axis/minor axis) were acquired by using ImageJ. Smaller values of AR and FF indicate increased mitochondrial fragmentation. *N*=17,714, 5,929 and 5,862 mitochondria in the groups of Vehicle, ISO 1 μM and ISO 10 μM, respectively. (**i**) Overexpression of CaMKII WT and CaMKII CA increased mitochondrial fragmentation in H9C2 cells. CaMKII WT potentiated the effects of ISO on mitochondrial morphological change as indicated by significant fragmentation at a low ISO dose (100 nM). *N*=17,714, 17,692, 9,567 and 17,035 mitochondria in the groups of Vehicle, CaMKII WT, WT+ISO and CaMKII CA, respectively. Data are mean±s.e.m. *P<0.05 versus Vehicle, ^#^*P*<0.05 versus ISO. The data were analysed using One-way ANOVA followed by Turkey post test.

CaMKII is a cytosolic kinase and we hypothesized that CaMKII may phosphorylate a cytosolic protein, which travels to mitochondria and modulates mPTP. Drp1 is a cytosolic protein that translocates to mitochondria upon activation by Ca^2+^ or phosphorylation^25^,^26^. A number of kinases can phosphorylate Drp1 at different sites^25^,^26^,^27^,^28^, such as by a CaMK family member, CaMKI at S637, in the brain^29^. Since *in vivo* phospho-proteomics analysis reveals proteins with serine/threonine were the cardiac targets of β-AR signalling^30^, we hypothesized that CaMKII may induce Drp1 phosphorylation and through which facilitates mPTP opening^11^. Indeed, by using site-specific phosphorylation antibodies, we found that 2 weeks of ISO infusion significantly increased Drp1 phosphorylation at S616 site (Drp1^S616^, 1.8 fold increase over control) in the mouse heart (Fig. 6a). Further, KN93 (10 μM kg^−1^) or β1-AR antagonist (propranolol, 10 mg kg^−1^), efficiently prevented Drp1^S616^ phosphorylation (Fig. 6a). In contrast, Drp1 phosphorylation at S637 site (Drp1^S637^) was only detected *in vitro* in ISO-treated (12 h) adult myocytes in a cAMP/PKA-dependent but CaMKII-independent manner as previously reported^25^, but not *in vivo* in the mouse heart after 2 weeks of ISO infusion (Supplementary Fig. 6a,b). In addition, CaMKII DN did not affect the phosphorylation status of Drp1 at S637 at baseline or after ISO treatment (Supplementary Fig. 6c), Overexpression WT Drp1 or S616A mutation equally increased S637 phosphorylation due to increased total Drp1 levels, suggesting the phosphorylation status at S616 has no effect on S637 phosphorylation (Supplementary Fig. 6d). In cultured adult cardiomyocytes, ISO treatment led to Drp1^S616^ phosphorylation as early as 6 h and can be abolished by KN93 (Fig. 6b,c). Further, overexpression of CaMKII WT promoted Drp1^S616^ phosphorylation (Fig. 6d). These results strongly suggest that CaMKII is upstream of Drp1^S616^ phosphorylation during chronic β1-AR stimulation.

Next, whether Drp1^S616^ phosphorylation promotes its translocation to mitochondria was determined by measuring Drp1 protein levels in mitochondrial and cytosolic fractions of the myocyte after 2 weeks of ISO infusion. Indeed, Drp1 was significantly enriched in mitochondrial fraction (1.84 fold) while decreased in cytosolic fraction (Fig. 6e). KN93 prevented these changes (Fig. 6e). In adult cardiomyocytes treated with ISO, increased ‘punctate' Drp1 pattern that colocalized with mitochondrial marker was found by immunofluorescent staining (Fig. 6f). Moreover, we found phosphorylated Drp1 (at S616 site) was enriched in the mitochondrial fraction of the heart preparations after 2 weeks of ISO infusion (Fig. 6g). These results provide strong support that Drp1 phosphorylation at S616 site promotes its mitochondrial translocation.

Finally, we tested whether the increased mitochondrial translocation of Drp1 affects mitochondrial morphology. In H9C2 cardiac myoblast cells, chronic ISO treatment (1 μM for 24 h) shifted the balance of fission/fusion towards fission as indicated by decreased aspect ratio (AR) and form factor (FF) (Fig. 6h). We have used these two parameters to quantify mitochondrial fission/fusion in other cells^31^. As positive control, we found that the dominant-negative Drp1 mutation, Drp1 K38A, increased AR and FF (Fig. 6h and Supplementary Fig. 7). Overexpression of CaMKII WT mildly increased, whereas overexpression of CaMKII CA significantly facilitated mitochondrial fission. CaMKII WT also enhanced ISO-induced mitochondrial fission (Fig. 6i and Supplementary Fig. 7). Calcineurin has been shown to dephosphorylate Drp1 at S637 site and promote its mitochondrial translocation^26^. Therefore, we tested whether CsA, also a calcineurin inhibitor, can affect Drp1 phosphorylation at S616 or S637 site and the mitochondrial translocation of Drp1 during ISO treatment. CsA had no effect on ISO-induced S616 and S637 phosphorylation in H9C2 cardiac myoblast cells, Drp1 translocation to mitochondria and mitochondrial fission (Supplementary Figs 7 and 8). Taken together, CaMKII promotes fission through Drp1 phosphorylation at S616 and its mitochondrial translocation.

### CaMKII bound Drp1 and directly phosphorylated it at S616

**Figure 7:**
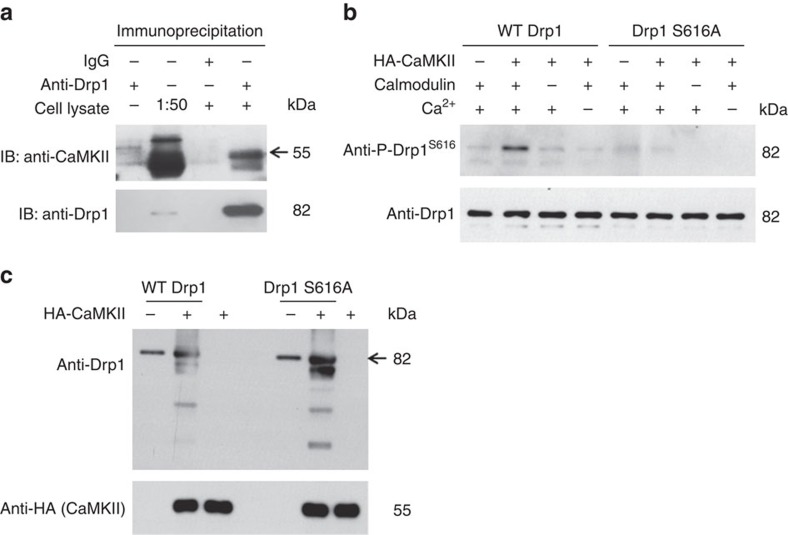
CaMKII binds and directly phosphorylates Drp1 at S616. (**a**) Co-immunoprecipitation analysis showing the binding of Drp1 with CaMKII in adult cardiomyocytes. Images are representative of four repeats. (**b**) HA-tagged CaMKII from H9C2 cells were attached to anti-HA magnetic beads and incubated with WT Drp1 or S616A mutation purified from *E. coli.* (2 μM) in the presence or absence of calmodulin (1 mM) and Ca^2+^ (1 mM) for 24 h. The supernatant was used for Western blot to detect Drp1 S616 phosphorylation. Images are representative of three repeats. (**c**) The beads were boiled and samples were used for Western blot using anti-HA (for CaMKII) or anti-Drp1 antibodies for determining the *in vitro* interaction between CaMKII and Drp1. Images are representative of three repeats.

CaMKII has many intracellular targets, including kinases and Drp1 S616 phosphorylation can be modulated by other kinases/phosphatases as well. Therefore, it is critical to determine whether CaMKII can directly bind and phosphorylate Drp1 at this site. Co-immunoprecipitation analysis showed that endogenous Drp1 and CaMKII in adult rat cardiomyocytes bound with each other (Fig. 7a). Moreover, we purified CaMKII from H9C2 cells and murine WT Drp1 or the S579A mutation (equivalent to human Drp1 S616A) from *Escherichia coli* (Supplementary Fig. 9) and incubated them together in the presence of calmodulin and Ca^2+^. The results showed that anti-P-S616 antibody detected phosphorylation in WT Drp1, but not the mutated Drp1, when incubated with CaMKII (Fig. 7b). Interestingly, in this *in vitro* phosphorylation reaction system, we found CaMKII and Drp1 S616A can form a more stable enzyme–substrate intermediate compared with CaMKII and WT Drp1 (Fig. 7c). Since, after being phosphorylated, the protein tends to dissociate with its kinase to facilitate subsequent binding and phosphorylation of a new protein, these results support that CaMKII directly binds and phosphorylates Drp1 at S616 site.

### Blocking Drp1 ameliorated myocyte and heart dysfunction

**Figure 8:**
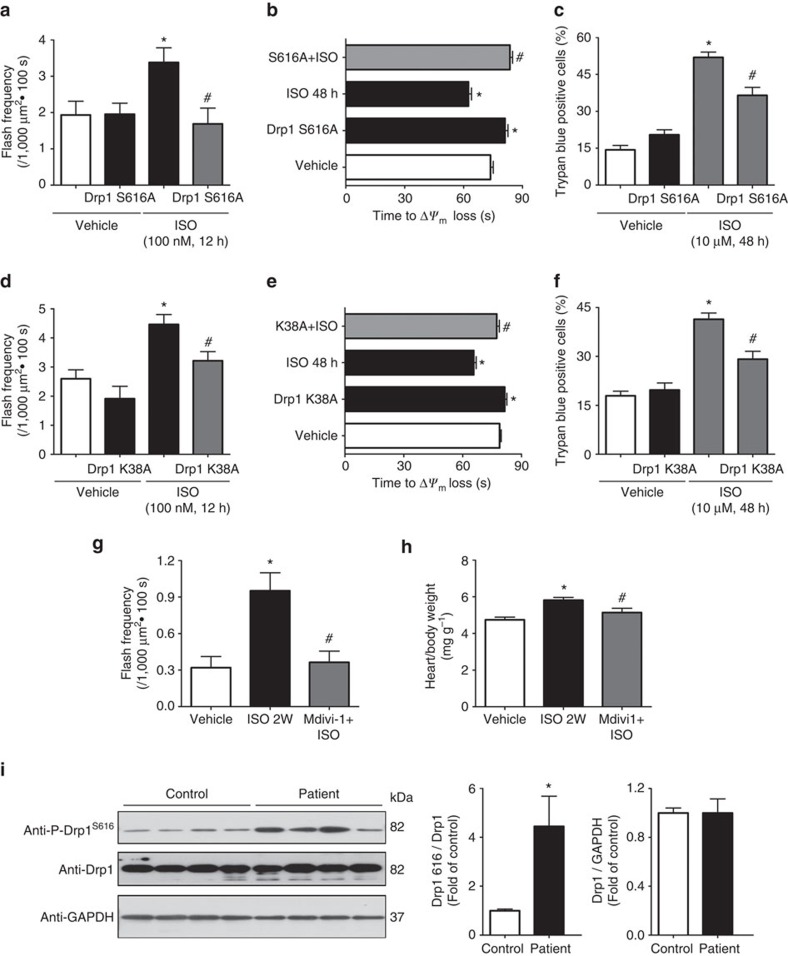
Drp1 inhibition suppressed ISO-induced mitochondrial and myocyte dysfunction and heart hypertrophy. (**a**) Preventing Drp1^S616^ phosphorylation by overexpression a phosphorylation null mutation of Drp1 (Drp1 S616A, in which serine at 616 was mutated to alanine) blocked ISO-induced flash frequency in adult cardiomyocytes. *N*=12, 14, 13 and 10 cells from three rats in the groups of Vehicle, Drp1 S616A, ISO and S616A+ISO, respectively. (**b**) Drp1 S616A also prolonged the time of laser-induced permanent loss of Δ*ψ*_m_ by chronic ISO treatment (1 μM, 48 h). *N*=193, 245, 231 and 224 mitochondria from 20 to 24 cells from three rats in the groups of Vehicle, Drp1 S616A, ISO and S616A+ISO, respectively. (**c**) Drp1 S616A rescued myocyte death induced by ISO treatment (10 μM, 48 h). *N*=1061, 985, 877 and 694 cells from three rats in the groups of Vehicle, Drp1 S616A, ISO and S616A+ISO, respectively. (**d**–**f**) Drp1 K38A also blocked ISO's effect on flash frequency (**d**), laser-induced permanent loss of Δ*ψ*_m_ (**e**) and myocyte death (**f**). In **d**, *N*=26, 11, 36, 17 cells; in **e**, *N*=479, 239, 198 and 196 mitochondria; and in **f**, *N*=536, 653, 697 and 819 cells in the groups of Vehicle, Drp1 K38A, ISO and K38A+ISO, respectively. (**g**) Mdivi-1 (50 mg kg^−1^ d^−1^), a chemical inhibitor of Drp1, efficiently attenuated the increased flash frequency in intact heart by 2 weeks of ISO infusion. *N*=25, 18 and 27 images from 4 to 6 hearts in the groups of Vehicle, ISO and Mdivi-1+ISO, respectively. (**h**) Mdivi-1 prevented ISO-induced cardiac hypertrophy. *N*=7–8 mice. Data in **a**–**h** are mean±s.e.m. *P<0.05 versus Vehicle group, ^#^*P*<0.05 versus ISO group. (**i**) Representative images and summarized data showing Drp1^S616^ phosphorylation and total Drp1 levels in the ventricular samples of heart failure patients. *N*=4 for each group. *P<0.05 versus None-failing control group. Data are mean±s.e.m. In **a**–**i**, the data were analysed using One-way ANOVA followed by Turkey post test.

Whether Drp1^S616^ phosphorylation contributes to CaMKII-induced mPTP is determined. We found that overexpression of non-phosphorylatable Drp1 mutation (Drp1 S616A) prevented ISO-induced flash activity (Fig. 8a). Moreover, cardiomyocytes overexpressing Drp1 S616A were resistant to laser-induced Δ*ψ*_m_ loss and showed less cell death after 48 h ISO treatment (Fig. 8b,c). Additionally, overexpressing Drp1 K38A prevented ISO-induced mPTP and cell death (Fig. 8d–f), CaMKII-induced mitochondrial flashes, and ISO's effect on laser-induced Δ*ψ*_m_ loss (Supplementary Fig. 10).

To gain therapeutic insights over the role of Drp1 in ISO-induced heart dysfunction, we blocked Drp1 activity *in vivo* by Mdivi-1 (50 mg kg^−1^), a compound inhibiting Drp1 activity, and found that ISO infusion-induced flash activity was decreased and cardiac hypertrophy attenuated (Fig. 8g,h). These results demonstrate that increased Drp1 activity likely due to Drp1^S616^ phosphorylation contributes to heart hypertrophy.

### Increased Drp1^S616^ phosphorylation in human failing hearts

Chronically elevated β1-AR activity is a hallmark of heart failure in human. To further test the clinical relevance of the CaMKII–Drp1–mPTP pathway, we determined Drp1^S616^ phosphorylation in human failing hearts. Ventricular samples from dilated cardiomyopathy or ischaemic heart failure patients^32^ showed significantly increased Drp1^S616^ phosphorylation with no change in total Drp1 levels (Fig. 8i). These data strongly support that increased Drp1^S616^ phosphorylation is associated with human heart failure.

## Discussion

**Figure 9:**
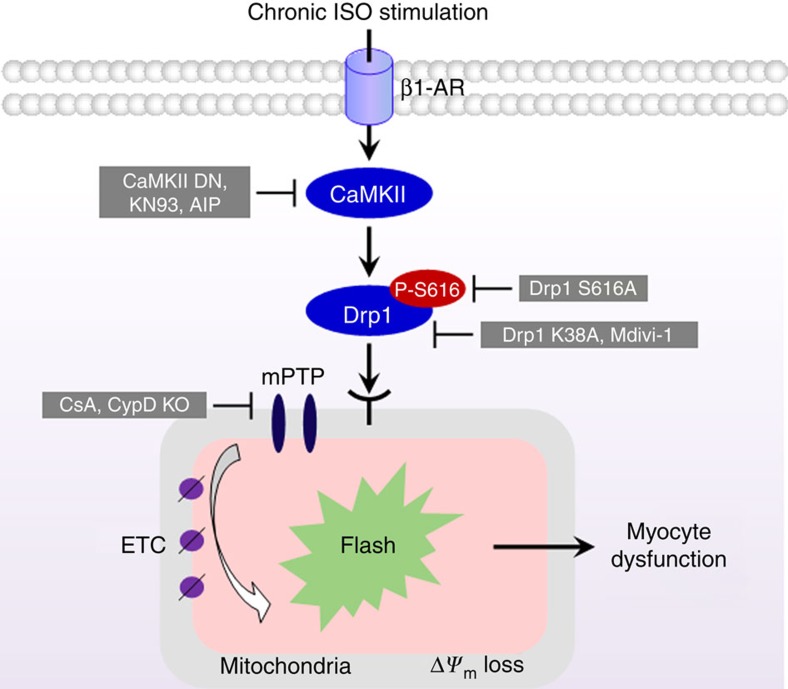
Schematic model of CaMKII mediating mPTP through Drp1^S616^ phosphorylation during chronic β-AR stimulation. Sustained ISO treatment activates CaMKII pathway, a downstream kinase of β1-AR, and subsequently increases the phosphorylation of Drp1 at S616 (Drp1^S616^), which activates Drp1. After translocating to the outer membrane of mitochondria, the phosphorylated Drp1 triggers fission and mPTP openings, which are recorded by mitochondrial flash events. Finally, chronic activation of this pathway leads to mitochondrial and myocyte dysfunction. Abolishing CaMKII activity (CaMKII DN, KN93 or AIP), inhibiting Drp1 activity (Drp1 K38A or Mdivi-1), preventing Drp1^S616^ phosphorylation (Drp1 S616A), or blocking mPTP openings (CsA or CypD KO) efficiently prevented myocyte damage and cardiac hypertrophy during chronic β1-AR stimulation.

In this report, we demonstrate a novel signalling pathway that mediates chronic β-AR stimulation-induced mitochondrial dysfunction (Fig. 9). Specifically, CaMKII directly phosphorylates a fission protein, Drp1 at a novel site and this post-translational modification enhances Drp1 translocation to mitochondria, mPTP openings and eventually myocyte death. Massive or irreversible mPTP openings are final executors of mitochondrial pathway of cell death and have been linked to chronic β-AR stimulation^6^,^33^. The mPTP is also known to open transiently or in subconductance state under physiological conditions^8^,^9^, which may bear essential signalling roles^34^,^35^. It is largely unknown whether these two modes of mPTP openings are connected and how the pathological mPTP is induced in the stressed heart. Results from this study show that the physiological mPTP openings, if activated persistently, are detrimental. Further, the CaMKII–Drp1–mPTP pathway could be a key modulator of stress-induced heart pathology and a potential target for heart failure therapy.

mPTP is a non-specific and large pore that can be triggered by pathological perturbations^9^,^10^,^11^,^33^. In the heart, massive and irreversible mPTP openings are the culprit of ischaemia reperfusion injury^9^. However, transient or subconductance mPTP openings are detected under resting conditions^36^,^37^, and may have important physiological functions^38^. Several pieces of evidence shown here suggest that the physiologically relevant mPTP openings (for example, mitochondrial flash-coupled) could be detrimental under stress conditions. mPTP openings was persistently increased after 12–18 h of ISO treatment and preceded oxidative stress, loss of Δ*ψ*_m_ and myocyte death. Blocking the persistent mPTP openings at the early phase (12 h) attenuated mitochondrial damage and myocyte death at the later stage. Increased mPTP was downstream of CaMKII pathway, which has been linked to cell death during chronic β-AR stimulation^4^,^5^,^7^,^19^. Finally, increased mPTP was observed in the heart after chronic ISO infusion. Thus, mPTP openings, if persistently activated, could induce mitochondrial and myocyte dysfunction.

How to differentiate the physiological and pathological mPTP openings is an important question. The mitochondrial flash is such an optical readout that may be used to answer this question. We have shown that flash-coupled mPTP in the normal heart is at a low frequency and is moderately induced by physiological stimulations such as Ca^2+^ transients during excitation–contraction coupling^22^. On the other hand, dramatic increases in flash-coupled mPTP openings such as by ischaemia reperfusion, excessive oxidants, or persistently high Ca^2+^, are detrimental^12^,^17^. Therefore, the magnitude and duration of mPTP openings could determine the eventual outcome. This study supports this idea by showing that chronic β-AR stimulation persistently increased the frequency of mPTP openings, and over time, caused myocyte death. It is conceivable that β-AR stimulation-induced mPTP openings may be a compensatory response to either stimulate mitochondrial respiration or limit matrix Ca^2+^ overload in the short term. However, in the long run, persistently increased mPTP openings could compromise bioenergetics, induce oxidative stress and disturb Ca^2+^ homeostasis. In agreement with this speculation, increased mPTP openings have been linked to oxidative insult-induced apoptosis and neural excitotoxicity^17^,^39^.

This study also provides evidence to show the causal role of CaMKII in chronic β-AR-induced mPTP openings and cell death. Chronic β-AR stimulation causes mitochondrial swelling, irreversible mPTP openings and mitochondrial-dependent apoptosis^6^,^33^. Whether these effects are mediated by CaMKII is not known. Here, we show that CaMKII activation is both sufficient and necessary to stimulate mPTP. First, ISO-induced mPTP openings was blocked by CaMKII inhibitors and a dominant-negative CaMKII mutation. Moreover, suppressing or enhancing CaMKII activity prevented or facilitated mPTP openings, mitochondrial damage and myocyte death. CaMKII also modulates cytosolic and sarcoplasmic reticulum (SR) Ca^2+^ handling and gene expression through phosphorylating various targets^40^,^41^. Specifically, the cytosolic Ca^2+^ derangement, such as increased SR leak and resting Ca^2+^ levels, may synergistically stimulate mPTP opening and contribute to cell death. This speculation is consistent with recent reports underscoring the role of CaMKII in mitotoxicity, heart dysfunction and hypertrophy^7^,^42^. A recent report showed that CaMKII knockout mouse has lower myocyte death but still develops heart hypertrophy after chronic ISO infusion^43^. We speculate that, unlike the pharmacological and acute inhibition of CaMKII in this study, total ablation of CaMKII in the heart may activate compensatory mechanisms that mediate the hypertrophy phenotype.

To address how CaMKII signalling pathway modulates mPTP, we show that mitochondrial fission protein Drp1 is a novel mediator. Drp1 is a cytosolic protein that can be activated/inactivated by phosphorylation at various sites^25^,^26^,^27^,^28^. It is not known whether CaMKII can phosphorylate Drp1. We detected increased Drp1 phosphorylation at S616 site during chronic ISO treatment or by CaMKII overexpression. Furthermore, CaMKII directly interacted with Drp1 and promoted its mitochondrial translocation and mitochondrial fission. These results are consistent with previous report showing Drp1^S616^ phosphorylation enhances Drp1 translocation and function^44^. Previous studies have also shown that acute ISO treatment induces Drp1^S637^ phosphorylation in a PKA-dependent manner^25^. It is possible that the phosphorylation of Drp1 at PKA site (S637) versus CaMKII site (S616) serves as a switch or checkpoint to determine distinct outcomes during acute versus chronic β-AR stimulation.

Finally, we show that Drp1^S616^ phosphorylation or Drp1 activity is required for β-AR stimulation- or CaMKII-induced mPTP openings, since mutating this phosphorylation site (S616A) or the GTP-binding site (K38A) of Drp1 efficiently blocked increased mPTP openings, mitochondrial damage and cardiomyocyte death. However, how Drp1 S616 phosphorylation or Drp1 activity stimulates mPTP is not fully understood. Previous reports indicates that mitochondrial fission is essential for mPTP openings in response to stresses such as hyperglycaemia, and inhibiting mitochondrial fission protects against ischaemia reperfusion injury probably through suppressing mPTP (refs 11, 31). Another possibility is that Drp1 may promote mitochondrial fission, which could cause membrane fractures or form lipidic pores^45^. Finally, the mitochondrial outer membrane proteins, such as the Bcl-2 family proteins, may interact with Drp1 and modulate mPTP (for example, through outer membrane permeability)^46^,^47^,^48^.

In summary, two novel findings were reported in this study. First, chronic β-AR stimulation persistently increased mPTP openings through CaMKII pathway. Second, CaMKII directly interacted and phosphorylated Drp1 at S616 site and through which increased mPTP openings. Therefore, activation of the cytosolic kinase CaMKII modulates mitochondrial function through phosphorylation of a fission protein. This pathway could contribute to mitochondrial dysfunction and myocyte death during chronic β-AR stimulation, a clinically important condition often seen in heart failure patients.

## Methods

### Reagents

ISO, CsA, CGP 20712A (CGP), ICI-118,551 (ICI), PKA inhibitor fragment 14–22 (PKI), RP-8-PIP-CAMPS (8-RP-cAMPs), AIP, (±)-propranolol hydrochloride (Prop), Mdivi-1, atractyloside and mitoTEMPO were purchased from Sigma-Aldrich (St Louis, MO, USA). KN93 and KN92 were purchased from Merck Millipore Corporation (La Jolla, CA, USA). The fluorescent dyes, tetramethylrhodamine methyl ester perchlorate (TMRM), Fluo-4 AM, DCFH-DA, MitoSOX red MitoTracker Red CMXRos and MitoTracker Green FM were purchased from Life Technologies (Eugene, OR, USA).

### Recombinant adenovirus vectors construction

Construction of recombinant adenovirus vectors containing: mitochondrial-targeted circularly permuted yellow fluorescent protein (Ad-mt-cpYFP)^12^, HA-tagged CaMKIIδ_C_ DN, CAMKIIδ_C_ WT and CaMKIIδ_C_ CA were generated previously^19^. Recombinant adenovirus expressing MCU short hairpin RNA and Drp1 S616A were generated by Vector BioLabs (Malvern, PA, USA) with the original construct obtained from Addgene (Cambridge, MA, USA). Adenovirus containing Drp1 K38A or mtHyper were generated previously^31^,^49^. Concentration of the viruses was determined to be at ∼1 × 10^11^ viral particles per ml. All the viruses were aliquoted and stored at −80 °C.

### Adult cardiomyocyte culture and gene transfer

All the animal procedures were approved by the Institutional Animal Care and Use Committee at the University of Washington. Cardiomyocytes were isolated from the heart of female Sprague Dawley rats (200–250 g, Harlan) using standard enzymatic technique^22^. Briefly, rat was anaesthetized by intraperitoneal (i.p.) injection of pentobarbital. The heart was quickly removed, cannulated via the ascending aorta, and mounted on a modified Langendorff perfusion system. The heart was perfused with oxygenated Krebs–Henseleit Buffer (KHB) solution supplemented with collagenase II (Worthington, USA) and hyaluronidase (Sigma, USA) at 37 °C. Rod shaped adult cardiac myocytes were collected and plated at a density of ∼2 × 10^4^ cells per coverslip precoated with 20 μg ml^−1^ laminin (Life Technologies, USA). The cells were cultured in serum-free M199 medium (Sigma) supplemented with 10 mM glutathione, 26.2 mM sodium bicarbonate, 0.02% bovine serum albumin and 50 U ml^−1^ penicillin–streptomycin. Adenovirus-mediated gene transfer was implemented 2 h after cell plating and at a multiplicity of infection of 50–100. The chemicals ISO, CsA and KN93 were added into culture medium at 24–36 h and the experiments were done 2–4 days after gene transfer. For sustained β-AR stimulation, cardiomyocytes were treated with various concentrations of ISO (100 nM, 1 μM and 10 μM) for various periods (6, 12, 24 and 48 h) with fresh ISO provided every 24 h. Chemicals including CGP, ICI, PKI, 8-RP-cAMPs, AIP, KN-93, KN-92 and atractyloside were added 0.5 h before ISO treatment. mitoTEMPO was added 1 h before ISO treatment.

### Animal experiments

Pan-tissue mt-cpYFP transgenic mice were generated using C57BL/6 mice (Charles River) and with the pUC-CAGGS-mt-cpYFP vector as previously reported^22^,^50^,^51^. The mice were housed under standard conditions with a 12-h light–dark cycle and a constant temperature (22±2 °C). Food and water were provided *ad libitum*. To establish the cardiac hypertrophy model, transgenic mice of both genders at the age of 8–10 weeks (littermates) were randomly assigned to surgery or sham operation groups. ISO (15 mg kg^−1^ d^−1^) with or without KN93 (10 μM kg^−1^ d^−1^), Prop (10 mg kg^−1^ d^−1^) and Mdivi-1 (50 mg kg^−1^ d^−1^) were delivered by mini-osmotic pumps (2002, Alzet Corporation) implanted subcutaneously under anaesthesia with avertin (2.5% wt/vol, 15 μl g^−1^ body weight, i.p.), respectively. After 2 weeks, mice were euthanized and hearts were excised for perfused heart confocal imaging or weighed, and frozen in liquid nitrogen for Western blot and real-time PCR assay. In addition, male CypD KO mice and its WT controls (B6;129-Ppiftm1 J mol J^−1^, 3 months old) were purchased from the Jackson Laboratory. The adult cardiomyocytes isolating from these mice were cultured and treated with 100 nM ISO to determine the role of CypD in ISO-induced cell death.

### Confocal imaging of mitochondrial flashes

Single mitochondrial flash was detected by using a Zeiss LSM 510 Meta confocal microscope equipped with a 40 × 1.3 NA oil immersion objective^12^. For detecting mitochondrial flashes in adult cardiomyocytes, cells infected with Ad-mt-cpYFP virus were incubated in modified KHB buffer (138 mM NaCl, 3.7 mM KCl, 1.2 mM KH_2_PO_4_, 5 mM Glucose, 20 mM HEPES and 1 mM CaCl_2_) at room temperature. The cells were cultured on 25 mm coverslips and tightly attached to a custom designed perfusion chamber mounted on confocal microscope stage. Dual excitation images of mt-cpYFP were taken by alternating excitation at 405 and 488 nm and collecting emissions at >505 nm. Time-lapse *x*,*y* images were acquired at 1,024 resolution for 100 frames and at a sampling rate of 1 s per frame. During the scan, the spatiotemporal occurrence of flash is stochastic and no apparent laser or time-dependent induction of flashes was observed. In a subset of experiments, mitochondrial membrane potential indicator, TMRM (20 nM, Invitrogen) was loaded into cells and tri-wavelength imaging of mt-cpYFP and TMRM were taken by sequential excitation at 405, 488 and 543 nm, and emissions collected at 505–550, 505–550 and >560 nm, respectively^22^.

For imaging single mitochondrial flash activity in intact hearts from mt-cpYFP transgenic mice, the hearts were perfused with modified KHB buffer containing 0.5 mM pyruvate and 10 mM glucose at 37 °C and bubbled with 95% O_2_ and 5% CO_2_. The fluorescence acquisition plane was focused about 30 μm into the myocardium from the heart surface. Blebbistatin (10 μM) and gentle pressure were applied when taking time lapse two-dimensional (2D) images of the myocardium. In some experiments, TMRM (Δ*ψ*_m_ indicator, 100–500 nM) was included in the perfusion solution^50^.

### Laser-induced Δ*ψ*_m_ loss and determination of resting Δ*ψ*_m_

Laser-induced abrupt Δ*ψ*_m_ loss was used as an indication of the sensitivity of mitochondria to oxidative stress-induced irreversible mPTP opening in intact myocytes as previously described^52^. Briefly, the adult cardiomyocytes were incubated with TMRM (20 nM) in KHB buffer at 37 °C for 20 min. Confocal linescan (*x*, *t*) was set-up along the long axis of the myocytes and encompass 10–15 mitochondria that showed clear pattern and TMRM loading. TMRM was excited at 543 nm and at a slow speed (35 ms per line) to trigger oxidative stress and Δ*ψ*_m_ loss, which is shown as a sudden loss of TMRM signal in that mitochondrion. The time from the start of the scan to the complete loss of TMRM signal was calculated as the time of mPTP openings. A shorter mPTP time indicates that the pore is more sensitive to laser-induced openings. The intensity of the TMRM fluorescence at beginning of the scan was used as the basal Δ*ψ*_m_ in individual mitochondrion.

### Cellular oxidative stress measurement

The determination of intracellular oxidant stress was based on the oxidation of DCFH-DA. Briefly, adult cardiomyocytes were incubated with CM-H2DCFDA (Invitrogen) at 37 °C for 20 min. Confocal imaging was taken by 488 nm excitation and emission was collected at >505 nm. Time lapse 2D images were taken and the rate of DCF-DA fluorescence change over 30 frames (1 s per frame) (d*F*/d*t*) was used as an indication of the cellular oxidative stress. For determining mitochondrial ROS production, we used MitoSOX red (10 μM, loading for 10 min at 37 °C) with 405 and 514 nm excitation and emissions collected at >530 nm. The mitochondrial-targeted H_2_O_2_ indicator, mtHyper, was excited at 405 and 488 nm and emission collected at >505 nm.

### Ca^2+^ transient and myocyte contraction

Measurements of Ca^2+^ transients and myocyte contraction were performed as previously reported^18^. After loading with the Ca^2+^ indicator Fluo-4 AM (Invitrogen, 2 μM for 30 min) in KHB buffer, adult cardiomyocytes were electrically stimulated locally (40 V, 1 Hz) by placing the extracellular electrodes close to the cell of interest. Ca^2+^ transients and cell shortening were measured with a confocal laser scanning microscope (LSM510, Carl Zeiss). Digital image analysis used customer designed programs coded in interactive data language^18^.

### Cell death determination

The effects of chronic β-AR stimulation on cell death was detected by trypan blue exclusion assay and CellEvent Caspase-3/7 Green Detection Reagent (Invitrogen). In trypan blue exclusion, cardiomyocytes were incubated with trypan blue (0.4% in PBS) for 3 min at room temperature. Images were taken within 3–5 min of trypan blue washout by a light microscope. Unstained cells (clear) were defined as viable and cells with blue colour were damaged. At least 50 cells were counted in each image and four images for each sample. To determine apoptosis in live cells, adult cardiomyocytes were incubated with CellEvent Caspase-3/7 Green Detection Reagent (Invitrogen) for 30 min at 37 °C according to the manufacturer's instruction. Fluorescence images were taken by a confocal microscope with excitation/emission wavelength at 488 nm/525 nm (Leica TCS SP8).

### Western blot analysis

The heart tissues and adult cardiomyocytes were lysed with cell lysis buffer in the presence of a cocktail of proteinase/phosphatase inhibitors (Cell Signaling Technology, Inc., Danvers, MA, USA) and centrifuged at 12,000*g* for 30 min at 4 °C. Isolation of mitochondrial and cytosolic fractions from whole heart was performed using the Mitochondria/cytosol Fractionation Kit (Beyotime Institute of Biotechnology, Shanghai, China). The human man heart samples were collected in a previous study^32^ and the study protocol was approved by local ethics committee. The extracted proteins were separated by NuPAGE Novex 4–12% Bis–Tris Gels (Life Technologies). After transferring the proteins on to nitrocellulose membranes or polyvinylidene difluoride (PVDF) membranes, the membranes were blocked and incubated with various primary antibodies at 4 °C overnight. We used the following primary antibodies for the Western blots: anti-MCU antibody (1:500, Sigma-Aldrich), anti-HA antibody (1:1,000, Covance Research Products Inc.), anti-Phospho-Drp1 (S616) and anti-Phospho-Drp1 (S637) antibodies (1:500, Cell Signaling Technology), anti-Drp1 antibody (1:500, BD Biosciences or Abcam), anti-Phospholamban Phospho Threonine-17 (1:5,000, Badrilla Ltd. Leeds, United Kingdom), anti-PGC-1α (1:1,000, Sigma-Aldrich), anti-Tfam (1:1,000, Cell Signaling Technology), anti-LC3 (1:1,000, Sigma-Aldrich), anti-P62 (1:500, Cell Signaling Technology), anti-Atg5 (1:500, Cell Signaling Technology), anti-COX IV (1:1,000, Abcam, Cambridge, MA, USA) and anti-β-actin (1:500, Abcam, Cambridge, MA, USA). The appropriate anti-rabbit or anti-mouse secondary antibodies were applied. The immunoblots signals were detected and quantified using an Odyssey Infrared Imaging System (LI-COR, Lincoln, NE, USA) or a ChemiDoc XRS+System with Image Lab Software (Bio-Rad). All the uncropped scannings are provided in Supplementary Fig. 11.

### Real-time PCR analysis

Quantitative real-time PCR was applied to detect the hypertrophy-related genes by using SYBR green (Bio-Rad). Total ribonucleic acid was isolated from fresh heart tissue using the RNeasy Kit, and reverse transcripted to complementary DNA by using Omniscript reverse synthase (Qiagen). The primers for ANP and BNP are kindly gift from Dr Stephen C. Kolwicz (University of Washington). The primers for ANP were 5′-ATTGACAGGATTGGAGCCCAGAGT-3′ and 5′-TGACACACCACAAGG GCTTAGGAT-3′; for BNP were 5′-GCCAGTCTCCAGAGCAATTCA-3′ and 5′-GGGCCATTTCCT CCGACTT-3′. The real-time PCR result for the messenger RNA levels of each gene was repeated four times and was normalized to 18S ribosomal RNA levels.

### Immunofluorescence detection of Drp1 distribution

To analyse the translocation of Drp1 into mitochondria, after being incubated with MitoTracker Red CMXRos probe (200 nM for 30 min at 37 °C ), adult cardiomyocytes or H9C2 cells were fixed with 4% (w/v) paraformaldehyde in PBS at 4 °C for 20 min. The cells were then permeabilized with 0.5% Triton X-100 in PBS for 15 min at room temperature. The cells were washed twice with PBS before being blocked with normal goat serum at 37 °C for 1 h. Subsequently, the cells were incubated with a mouse monoclonal antibody against Drp1 (BD Biosciences) at a 1:100 dilution overnight at 4 °C, and then with an Alexa Fluor 488 goat anti-mouse IgG (Invitrogen) at a 1:100 dilution for 1 h at 37 °C. The cells were mounted in mounting medium and visualized under a Leica TCS SP8 laser scanning confocal microscope.

### Mitochondrial morphology analysis in H9C2 cell line

The effects of chronic β-AR receptor stimulation on mitochondrial morphology were carried out in a rat cardiac myoblast cell line (H9C2 cells; American Type Culture Collection, Manassas, VA, USA). H9C2 cells were cultured in DMEM (Invitrogen) supplemented with 10% FBS (Gibco, Grand Island, NY, USA), 1% v/v penicillin/streptomycin (Sigma) in a 5% CO_2_-humidified atmosphere at 37 °C. To analyse the mitochondrial morphology changes, H9C2 cells were loaded with 200 nM MitoTracker Green for 30 min at 37 °C. Confocal 2D images were collected by excitation at 488 nm and collecting emissions at >505 nm. Quantitative analyses of mitochondrial morphology were performed by ImageJ software (NIH)^31^. Individual mitochondria (particles) were subjected to particle analyses to acquire values for the FF (the reciprocal of circularity value) and AR (major axis/minor axis). High values for FF represent elongated tubular mitochondria, and increased AR values indicate an increase of mitochondrial complexity (length and branching).

### Co-immunoprecipitation

Adult cardiomyocytes from rats were collected in PBS with 0.1% Triton X-100. After three freeze–thaw cycles, the cell lysates were centrifuged at 13,000 r.p.m. for 15 min. The supernatant were incubated with Protein A/G agarose beads (Thermo Fisher) for 2 h to remove IgG in the samples. The samples were centrifuged (5,000 r.p.m.) and the protein concentrations in the supernatant were determined by Pierce BCA protein assay kit. The protein samples were incubated with either mouse anti-Drp1 antibody (BD Biosciences, 611113) or mouse IgG (Sigma, A0919) for 12 h at 4 °C. The Protein A/G agarose beads were added and incubated overnight at 4 °C. After binding, the beads were washed with PBS containing 0.1% Triton X-100 for five times. Proteins were eluted by using 0.1 M glycine (pH=2.7) and loaded on SDS–polyacrylamide gel electrophoresis (PAGE) for Western blot. The antibody used are rabbit anti-Drp1 (Novus Biologicals, NB11055288, 1:2,000), rabbit anti-CaMKII (GeneTex, GTX111401, 1:1,000) and mouse anti-rabbit light chain second antibody (Jackson ImmunoResearch Laboratories, 1:10,000).

### *In vitro* Drp1 phosphorylation and binding assay

The complementary DNA of mouse WT Drp1 or the S579A (equivalent to S616A in human) mutation was cloned into pET30a vector between the Nde1 and Not1 sites (Novagen) by using the forward primer: 5′-CAT ATG CAC CAT CAT CAT CAT CAT GAG GCG CTG ATC CCG GTC ATC-3′, and the reverse primer: 5′-GCG GCC GCT CAT CAC CAA AGA TGA GTC TCT CGG ATT TCA-3′. The forward primer contains 6 × His tag sequence. *E. coli* Rosetta (DE3) cells were used for the prokaryotic expression of Drp1 proteins. When the cell density reached an OD_600_ of ∼1.0, protein expression was induced by adding 0.5 mM isopropyl 1-thio-β-D-galactopyranoside and the cells were cultured at 4 °C for 7 days. The resulting poly-His-tagged WT Drp1 or S579A mutation was purified by Nickle resin (Ni Sepharose 6 Fast Flow, GE Healthcare) followed by imidazole elution. The purified recombinant Drp1 or Drp1 S579A was evaluated by SDS–PAGE and coomassie blue staining which revealed a purity of ∼90% and a molecular weight of ∼80 kDa (Supplementary Fig. 9). HA-tagged CAMKIIδ_C_ WT was purified from H9C2 myoblast cells after adenovirus-mediated gene expression. Calmodulin was purchased from Millipore.

For *in vitro* Drp1 phosphorylation assay, anti-HA magnetic beads (Pierce) were used to concentrate and immobilize HA-CaMKII. The immobilized HA-CaMKII beads (5 μl) were incubated with purified Drp1 or Drp1 S616A (2 μM) in a 100 μl reaction buffer containing 50 mM Tris–HCl, 150 mM NaCl and 25 μM ATP (pH7.4) for 24 h at 30 °C in the presence or absence of 1 mM CaCl_2_ and/or 1 mM calmodulin. After the incubation, the supernatant was used to detect Drp1 phosphorylation by Western blots using anti-P-S616 antibody (1:1,000, Cell Signaling) and rabbit anti-Drp1 antibody (1:2,000, Cell Signaling). The beads were washed, and immobilized HA-CaMKII and Drp1 complex was eluted by boiling, and the samples were used for Western blot analysis using rabbit anti-HA (1:5,000, Sigma) and rabbit anti-Drp1 (1:2,000, Cell Signaling) antibodies.

### Statistical analysis

All experimental results are expressed as mean±s.e.m. Each experiment was conducted at least three times. When multiple experiments using different numbers of animals were pooled for the statistical analysis, the range of number of animals was indicated in the figure legend. Data comparisons among the groups were performed using One-way ANOVA and unpaired Student's *t*-test when appropriate. A *P* value <0.05 was considered statistically significant.

### Data availability

All data supporting the findings of this study are available within the article, its Supplementary Information File, or from the corresponding authors on request.

## Additional information

**How to cite this article:** Xu, S. *et al*. CaMKII induces permeability transition through Drp1 phosphorylation during chronic β-AR stimulation. *Nat. Commun.* **7,** 13189 doi: 10.1038/ncomms13189 (2016).

## Supplementary Material

Supplementary Information Supplementary Figures 1-11.
