# Supplementary material for: CaMKII induces permeability transition through Drp1 phosphorylation during chronic β-AR stimulation
Source: Nat Commun. 2016 Oct 14;7:13189. doi: 10.1038/ncomms13189 (PMC5067512; doi:10.1038/ncomms13189)
Supplement: Supplementary Information — Supplementary Figures 1-11. [file ncomms13189-s1.pdf]

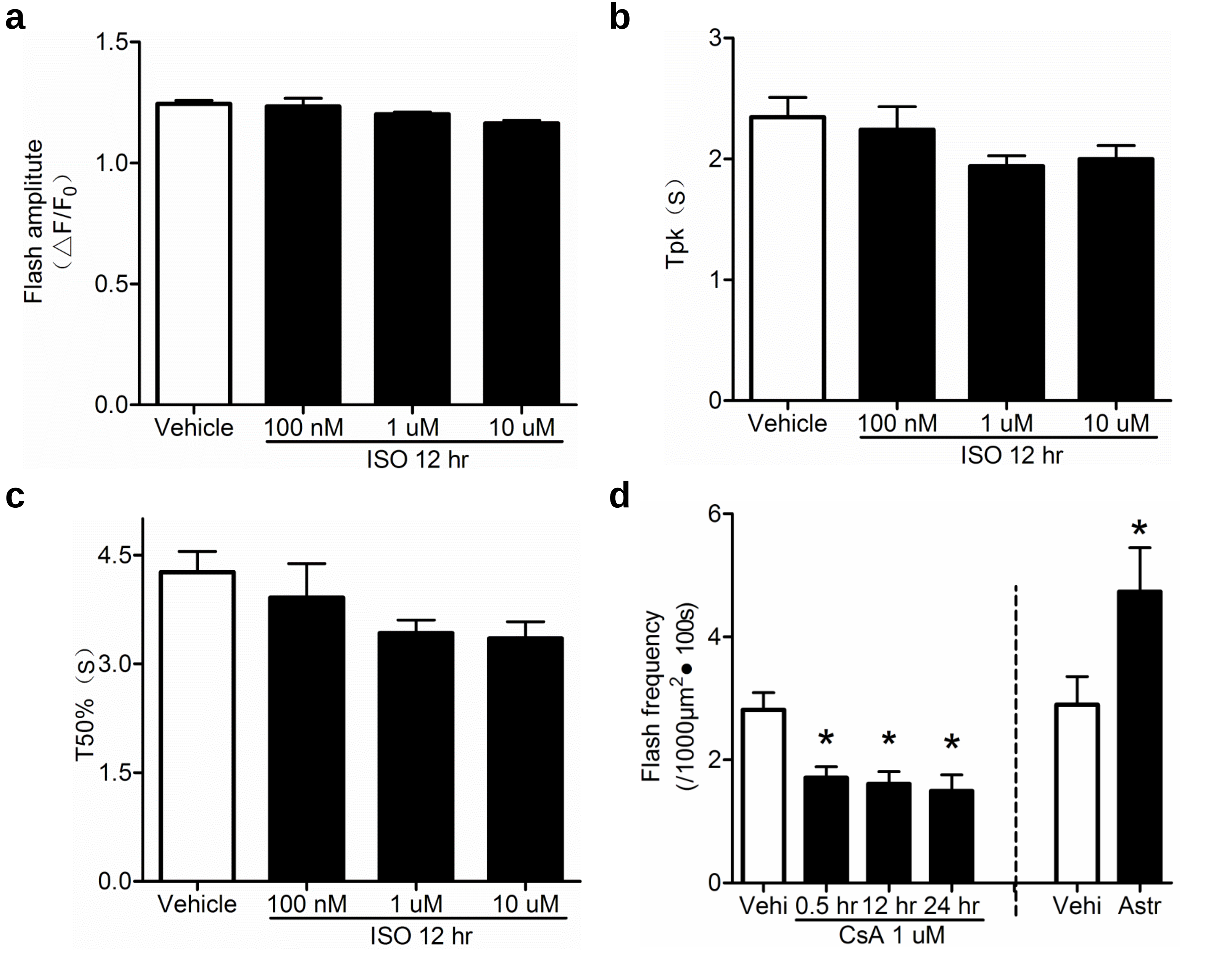
a
1.5
1.0
0.5
0.0
Flash amplitute
（△F/F0）
Vehicle
100 nM
1 uM
10 uM
ISO 12 hr
b
3
2
1
0
Tpk（S）
Vehicle
100 nM
1 uM
10 uM
ISO 12 hr
c
4.5
3.0
1.5
0.0
T50%（S）
Vehicle
100 nM
1 uM
10 uM
ISO 12 hr
d
6
4
2
0
Flash frequency
(/1000μm2● 100s)
*
*
*
*
Vehi
0.5 hr
12 hr
24 hr
CsA 1 uM
Vehi
Astr

**Figure S1**

**Supplementary Figure 1.** (**a–c**) Summarized data showing the properties of ISO (12 hr)-induced flashes, including amplitude (ΔF/F0, a), time to peak (Tpk, b) and time from peak to 50% decay (T50%, c), N = 165, 324, 141 and 84 flashes from 12-43 myocytes in the Vehicle, 100 nM, 1µM and 10 µM ISO groups, respectively. (**d**) Opposing effects of mPTP inhibition by CsA (1 µM for 0.5 hr, 12 hr, 24 hr, N = 25, 22, 11, and 13 cells respectively) or activation by atracyloside (20 µM, 0.5 hr, N = 11 cells) on superoxide flash frequency. Data in (**a-d**) are mean ± s.e.m. *P<0.05 versus Vehicle group. The data were analyzed using One-way ANOVA followed by Turkey post-test in (**a-c**) and CsA part of (**d**), and Student's t-test in Astr part of (**d**)

.

**Figure S1**

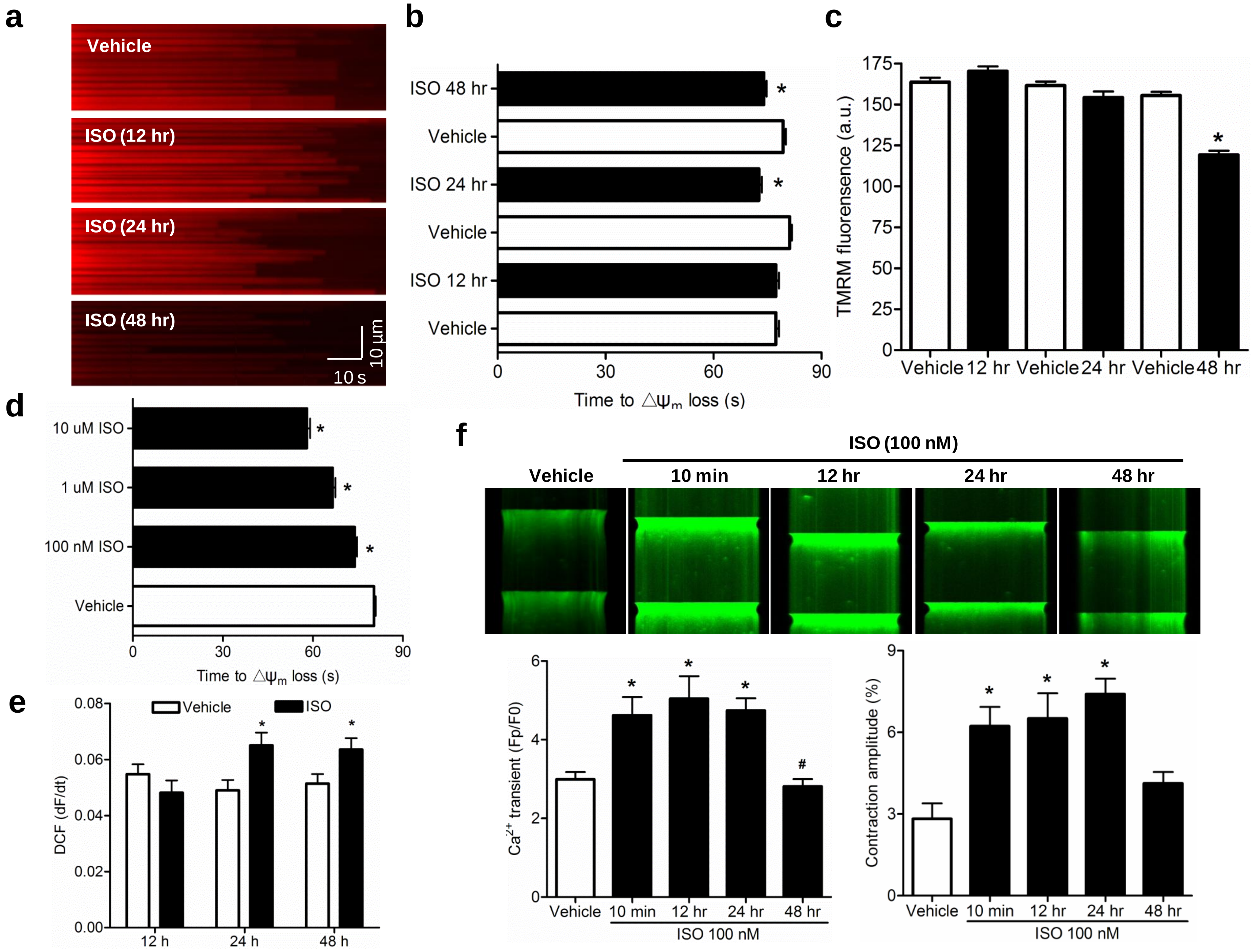
a
Vehicle
ISO (12 hr)
ISO (24 hr)
ISO (48 hr)
10 μm
10 s
b
ISO 48 hr
Vehicle
ISO 24 hr
Vehicle
ISO 12 hr
Vehicle
0
30
60
90
Time to △Ψm loss (s)
c
TMRM fluorensence (a.u.)
175
150
125
100
75
50
25
0
Vehicle
12 hr
Vehicle
24 hr
Vehicle
48 hr
d
10 uM ISO
1 uM ISO
100 nM ISO
Vehicle
0
30
60
90
Time to △Ψm loss (s)
e
DCF (dF/dt)
0.08
0.06
0.04
0.02
0.00
Vehicle
ISO
12 h
24 h
48 h
f
ISO (100 nM)
Vehicle
10 min
12 hr
24 hr
48 hr
Ca2+ transient (Fp/F0)
6
4
2
0
#
Vehicle
10 min
12 hr
24 hr
48 hr
ISO 100 nM
Contraction amplitude (%)
9
6
3
0
Vehicle
10 min
12 hr
24 hr
48 hr
ISO 100 nM

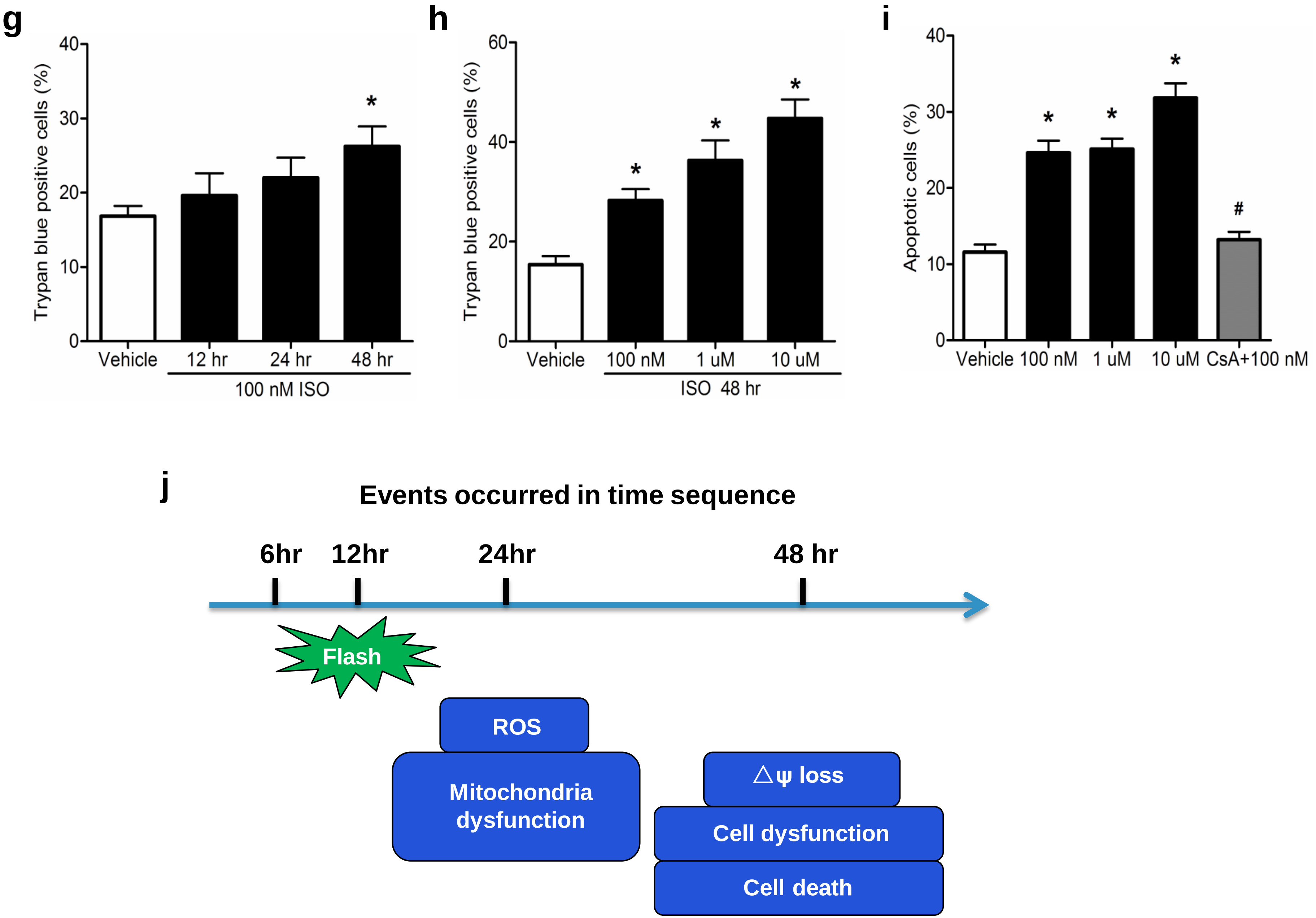
g
Trypan blue positive cells (%)
40
30
20
10
0
*
Vehicle
12 hr
24 hr
48 hr
100 nM ISO
h
Trypan blue positive cells (%)
60
40
20
0
*
*
*
Vehicle
100 nM
1 uM
10 uM
ISO 48 hr
i
Apoptotic cells (%)
40
30
20
10
0
*
*
*
#
Vehicle
100 nM
1 uM
10 uM
CsA+100 nM
j
Events occurred in time sequence
6hr
12hr
24hr
48 hr
Flash
ROS
Mitochondria dysfunction
△ψ loss
Cell dysfunction
Cell death

**Figure S2**

**Supplementary Figure 2.** Chronic β-AR stimulation led to mitochondrial and myocyte dysfunction in adult cardiomyocytes. (**a**) Representative linescan confocal images showing the laser-induced loss of $\triangle\psi_m$ by ISO (100 nM for 12-48 hr). (**b**) Quantification of the time from the start of scan to the sudden loss of $\triangle\psi_m$ in adult cardiomyocyts treated with ISO (100 nM for 12-48 hr). In Vehicle group, N = 290, 488, and 389 mitochondria from 23-39 cells in the time points of 12, 24, and 48 hr, respectively. In ISO group, N = 300, 388, and 463 mitochondria from 23-39 cells in the time points of 12, 24, and 48 hr, respectively. *$P<0.05$ versus Vehicle group. (**c**) The time course analysis of ISO on basal $\triangle\psi_m$ in individual mitochondrion. *$P<0.05$ versus Vehicle group. (**d**) ISO promoted the laser-induced loss of $\triangle\psi_m$ in a dose-dependent manner. N = 597, 463, 344, and 229 mitochondria in the groups of ISO 100 nM, 1µM, and 10 µM, respectively. *$P<0.05$ versus Vehicle group. (**e**) Time-course analysis for the effects of ISO (100 nM) treatment on cellular oxidative stress. *$P<0.05$ versus Vehicle group. N = 40, 23, 46, and 46 cells from 3 rats in the groups of Vehicle, 12 hr, 24 hr and 48 hr ISO, respectively.

**Figure S2**

(**f**) Prolonged ISO treatment reduced $Ca^{2+}$ transients and myocyte contraction. Upper panel: Linescan confocal images of $Ca^{2+}$ transients in cells subjected to ISO (100 nM) with local electrical stimulation (40 V, 1 Hz). N = 35, 23, 23, 31, and 49 cells in the groups of Vehicle, 10 min, 12 hr, 24 hr and 48 hr ISO, respectively. *P<0.05 versus Vehicle group, $^{\#}$P<0.05 versus ISO (10 min) group. (**g, h**) Trypan blue analysis showing time- and dose-dependent effects of ISO on myocyte death. In (**g**), N = 2038, 985, 1216, and 1079 myocytes in the groups of Vehicle, 12 hr, 24 hr and 48 hr ISO, respectively. In (**h**), N = 903, 585, 643, and 577 myocytes in the groups of Vehicle, 100 nM, 1µM and 10 µM, respectively. (**i**) Effects of ISO on myocyte apoptosis by measuring caspase-3/7 activity (CellEvent Caspase-3/7 Green Detection Reagent). CsA administration protected against ISO-induced apoptosis. N = 926, 957, 978, 955, and 937 myocytes from 4 rats in the groups of Vehicle, 100 nM, 1 µM, 10 µM ISO and CsA+100 nM, respectively. Data in (**b-i**) are mean ± s.e.m. (**j**) Diagram showing the sequential events of mitochondrial and myocyte dysfunction observed during chronic β-AR stimulation. The data were analyzed using by Student's t-test in (**b, c,** and **e**) and One-way ANOVA followed by Turkey post-test in (**d**, and **f-i**).

**Figure S2**

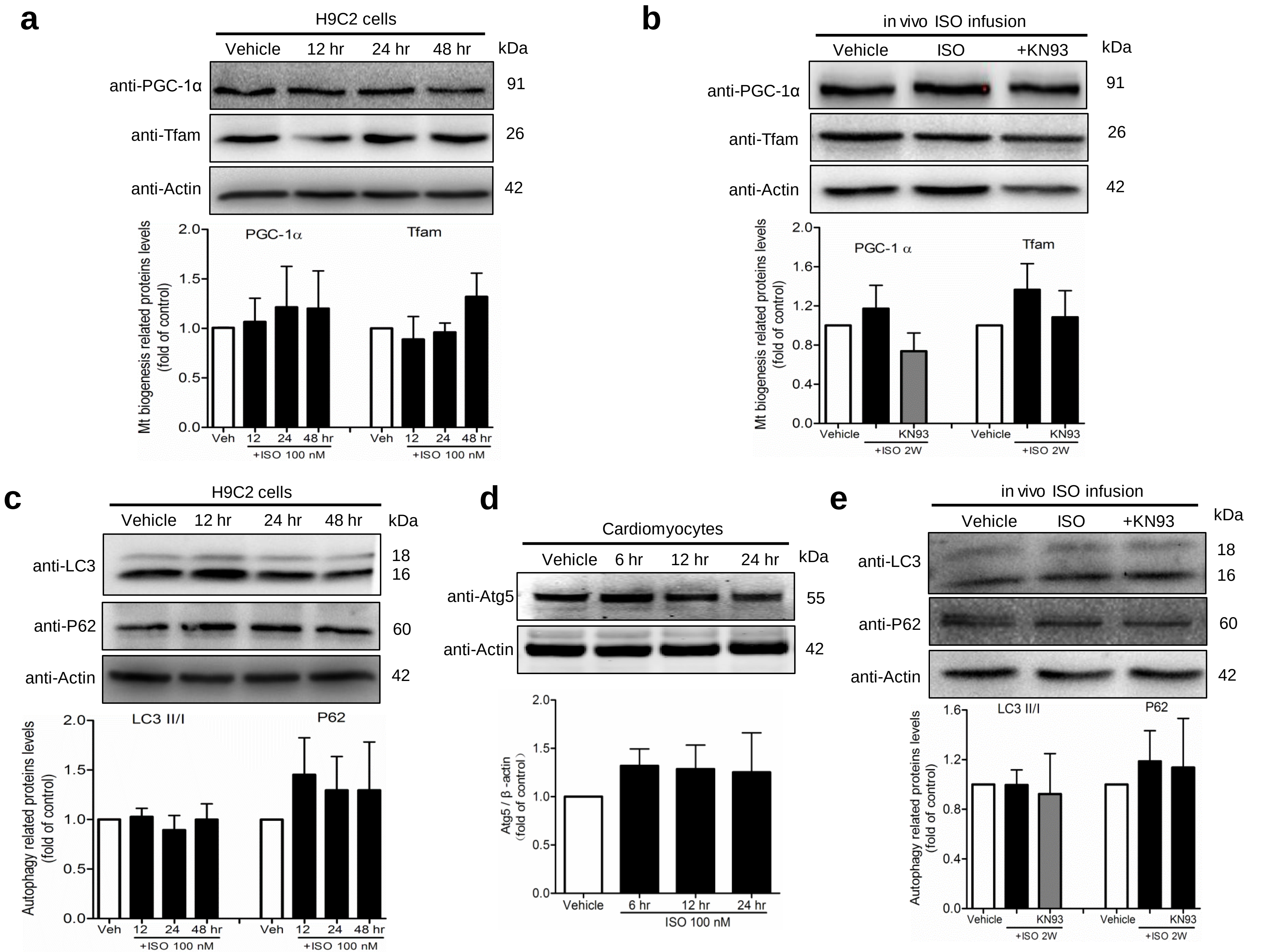
a
H9C2 cells
Vehicle
12 hr
24 hr
48 hr
kDa
anti-PGC-1α
91
anti-Tfam
26
anti-Actin
42
Mt biogenesis related proteins levels (fold of control)
PGC-1α
Tfam
2.0
1.5
1.0
0.5
0.0
Veh
12
24
48 hr
+ISO 100 nM
b
in vivo ISO infusion
Vehicle
ISO
+KN93
kDa
anti-PGC-1α
91
anti-Tfam
26
anti-Actin
42
Mt biogenesis related proteins levels (fold of control)
PGC-1 α
Tfam
2.0
1.6
1.2
0.8
0.4
0.0
Vehicle
KN93
+ISO 2W
c
H9C2 cells
Vehicle
12 hr
24 hr
48 hr
kDa
anti-LC3
18
16
anti-P62
60
anti-Actin
42
Autophagy related proteins levels (fold of control)
LC3 II/I
P62
2.0
1.5
1.0
0.5
0.0
Veh
12
24
48 hr
+ISO 100 nM
d
Cardiomyocytes
Vehicle
6 hr
12 hr
24 hr
kDa
anti-Atg5
55
anti-Actin
42
Atg5 / β -actin (fold of control)
2.0
1.5
1.0
0.5
0.0
Vehicle
6 hr
12 hr
24 hr
ISO 100 nM
e
in vivo ISO infusion
Vehicle
ISO
+KN93
kDa
anti-LC3
18
16
anti-P62
60
anti-Actin
42
Autophagy related proteins levels (fold of control)
LC3 II/I
P62
1.6
1.2
0.8
0.4
0.0
Vehicle
KN93
+ISO 2W

**Figure S3**

**Supplementary Figure 3.** Chronic ISO stimulation had no effect on mitochondrial biogenesis and autophagy. (**a-b**) Western blot determination of the protein levels of PGC-1α and Tfam, the key players in mitochondrial biogenesis, in H9C2 cell treated with ISO (**a**, 100 nM for 12-48 hr, N = 4) and mouse heart after ISO infusion (**b**, 15 mg/kg/day for 2 weeks, N = 5). (**c-e**) ISO stimulation had no effect on mitochondrial autophagy in H9C2 cells (**c**, 100 nM for 12-48 hr, N = 4), adult cardiomyocytes (**d**, 100 nM for 6-24 hr, N = 6) and mouse heart after ISO infusion (**e**, 15 mg/kg/day for 2 weeks, N = 5). Data are mean ± s.e.m. The data were analyzed by using One-way ANOVA followed by Turkey post-test in (**a-e**), no significant differences.

.

**Figure S3**

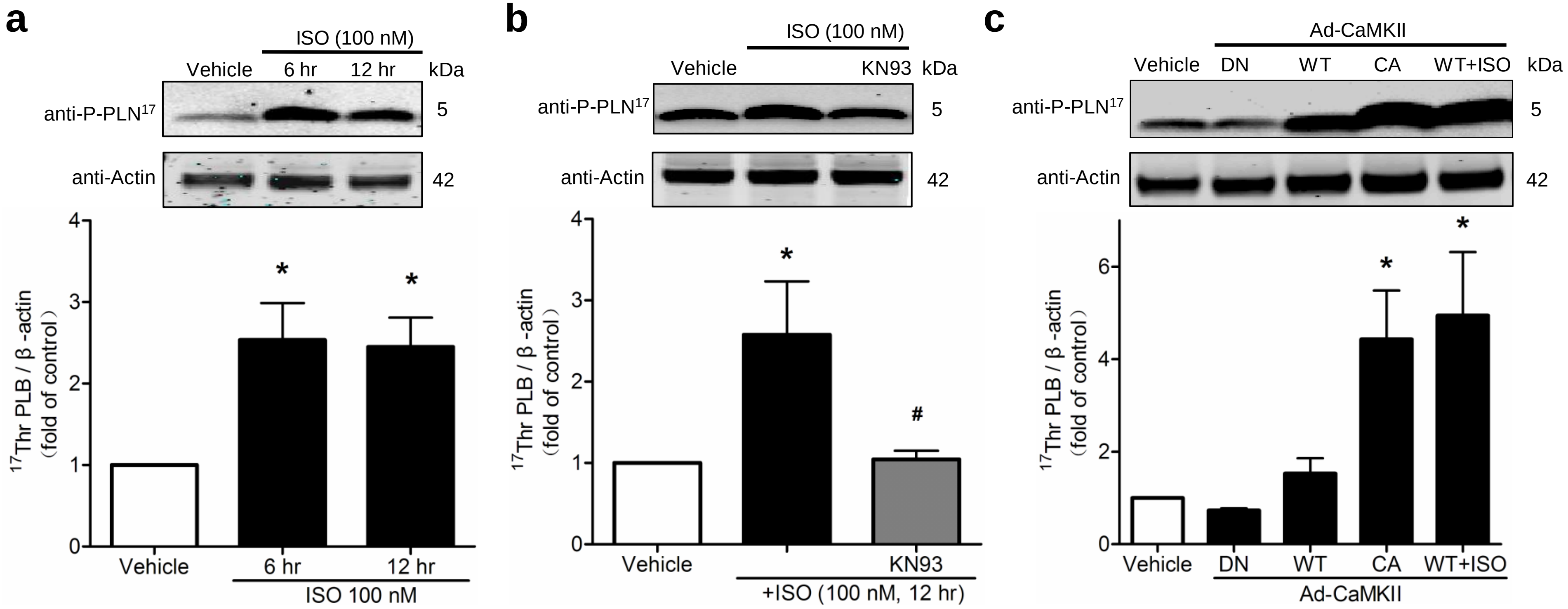
a
ISO (100 nM)
Vehicle
6 hr
12 hr
kDa
anti-P-PLN17
5
anti-Actin
42
17Thr PLB / β -actin (fold of control)
Vehicle
6 hr
12 hr
ISO 100 nM
b
ISO (100 nM)
Vehicle
KN93
kDa
anti-P-PLN17
5
anti-Actin
42
17Thr PLB / β -actin (fold of control)
Vehicle
KN93
+ISO (100 nM, 12 hr)
c
Ad-CaMKII
Vehicle
DN
WT
CA
WT+ISO
kDa
anti-P-PLN17
5
anti-Actin
42
17Thr PLB / β -actin (fold of control)
Vehicle
DN
WT
CA
WT+ISO
Ad-CaMKII

**Figure S4**

**Supplementary Figure 4.** Verification of the manipulation of CaMKII pathway by ISO or overexpression of CaMKII related mutations. (**a**) Western blot detected the phosphorylation of phospholamban (PLB) at Thr17, a known downstream site of CaMKII, in adult cardiomyocytes by ISO (100 nM for 6 hr and 12 hr). N = 3. (**b**) CaMKII blocker, KN93 prevented ISO-induced phosphorylation of PLB at Thr17 in adult cardiomyocytes. N = 3. (**c**) Adenovirus mediated overexpression of CaMKII constructs (DN, WT or CA) regulated the phosphorylation of PLB at Thr17 in adult cardiomyocytes. N = 3. Data in (**a-c**) are mean $\pm$ s.e.m. *P<0.05 versus Vehicle group. The data were analyzed by using One-way ANOVA followed by Turkey post-test in (**a-c**).

.

**Figure S4**

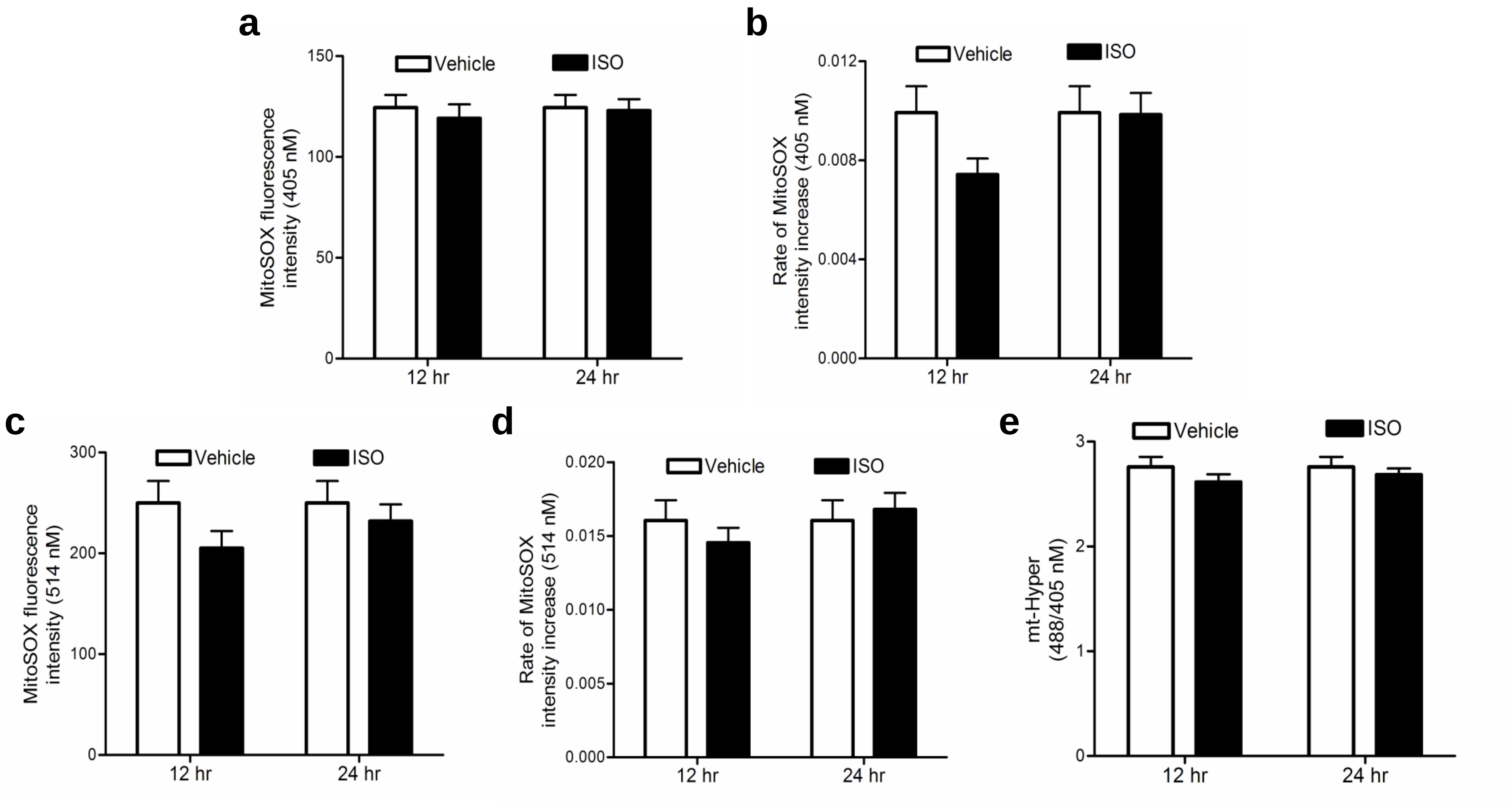
a
b
c
d
e
Vehicle
ISO
MitoSOX fluorescence intensity (405 nM)
Rate of MitoSOX intensity increase (405 nM)
MitoSOX fluorescence intensity (514 nM)
Rate of MitoSOX intensity increase (514 nM)
mt-Hyper (488/405 nM)
12 hr
24 hr

Figure S5

**Supplementary Figure 5.** Monitoring of mitochondrial ROS with MitoSOX red (**a-d**) or mitochondrial targeted $H_2O_2$ indicator (mtHyper) (**e**) during ISO (100 nM) stimulation. Data are mean $\pm$ s.e.m. In (**a-d**), N = 46, 38, 46, and 39 cells, and in (**e**), N = 26, 31, 26, and 41 cells from 3-4 rats in the groups of Vehicle 12 hr, 12 hr ISO, Vehicle 24 hr, and 24 hr ISO, respectively. The data were analyzed by using Student's t-test in (**a-e**). No significant differences.

**Figure S5**

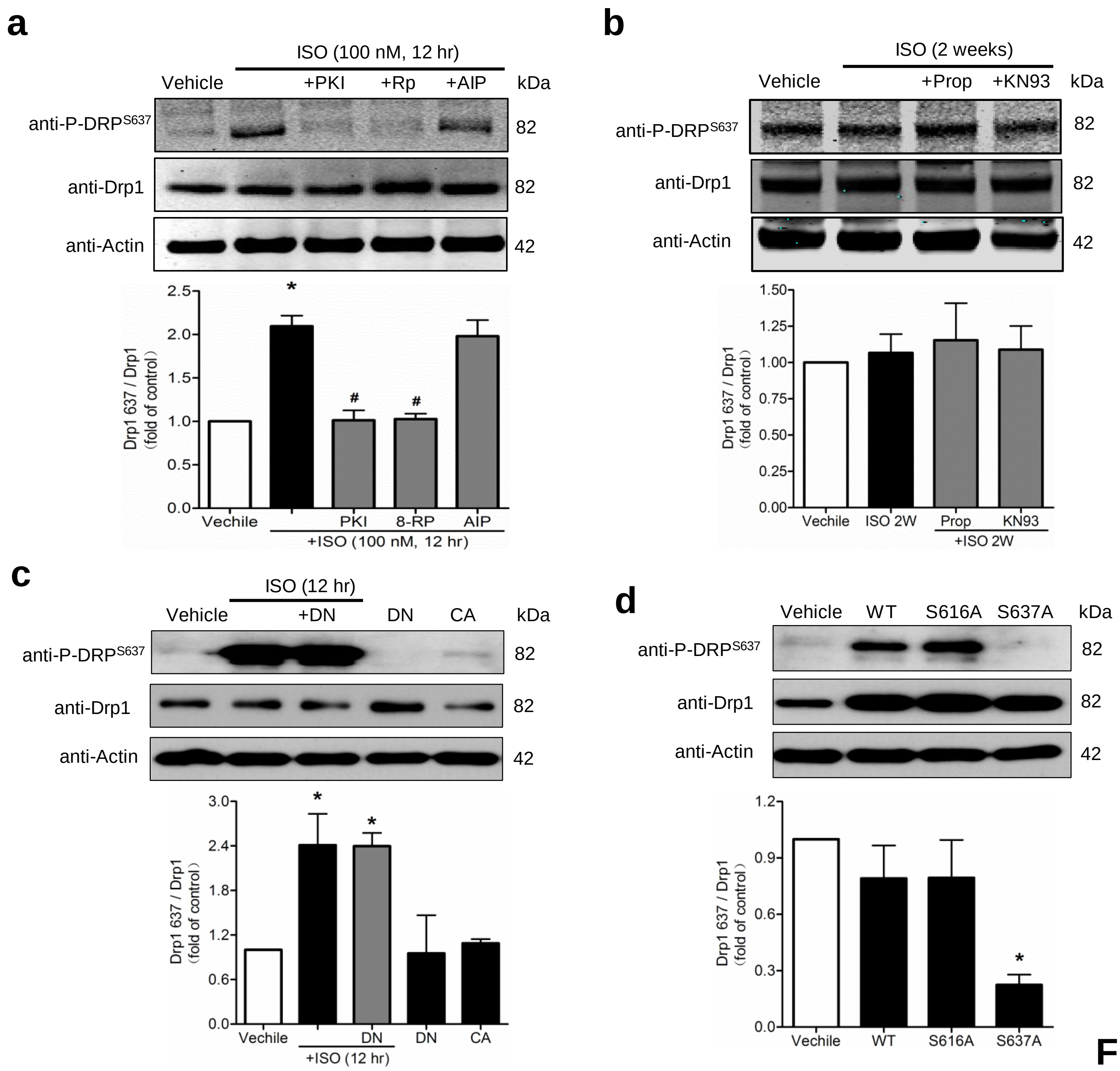
a
ISO (100 nM, 12 hr)
Vehicle
+PKI
+Rp
+AIP
kDa
anti-P-DRPS637
anti-Drp1
anti-Actin
82
82
42
Drp1 637 / Drp1 (fold of control)
Vechile
PKI
8-RP
AIP
+ISO (100 nM, 12 hr)
b
ISO (2 weeks)
Vehicle
+Prop
+KN93
kDa
anti-P-DRPS637
anti-Drp1
anti-Actin
82
82
42
Drp1 637 / Drp1 (fold of control)
Vechile
ISO 2W
Prop
KN93
+ISO 2W
c
ISO (12 hr)
Vehicle
+DN
DN
CA
kDa
anti-P-DRPS637
anti-Drp1
anti-Actin
82
82
42
Drp1 637 / Drp1 (fold of control)
Vechile
DN
DN
CA
+ISO (12 hr)
d
Vehicle
WT
S616A
S637A
kDa
anti-P-DRPS637
anti-Drp1
anti-Actin
82
82
42
Drp1 637 / Drp1 (fold of control)
Vechile
WT
S616A
S637A

**Figure S6**

**Supplementary Figure 6.** The phosphorylation of Drp1 at serine 637 was not involved in CaMKII-induced cardiotoxicity during the chronic ISO stimulation. (**a**) PKA blockers (PKI and 8-RP-cAMPs), not CaMKII blocker (AIP), attenuated the phosphorylation of Drp1 serine 637 in ISO-treated cardiomyocytes. N = 3. *P<0.05 versus Vehicle group, $^{\#}$P<0.05 versus ISO group. (**b**) Chronic ISO (15 mg/kg/day, 2 weeks) treatment did not induce phosphorylation of Drp1 at serine 637 site in the hypertrophic heart. N = 4. (**c**) Overexpression of CaMKII CA or CaMKII DN did not change the phosphorylation of Drp1 at serine 637. CaMKII DN also had no effect on ISO-induced Drp1 phosphorylation at serine 637 in adult cardiomyocytes. (**d**) Overexpression wild type Drp1 (WT) or S616A mutation similarly increased Drp1 phosphorylation at S637 likely due to increased total Drp1 levels. The overexpression of S637A mutation cannot be phosphorylated at this site and led to unchanged S637 phosphorylation levels in adult cardiomyocytes. Image in (**c**) and (**d**) are representative of 3 independent experiments. Data are mean ± s.e.m. *P<0.05 versus Vehicle group. The data were analyzed by using One-way ANOVA followed by Turkey post-test in (**a-d**).

**Figure S6**

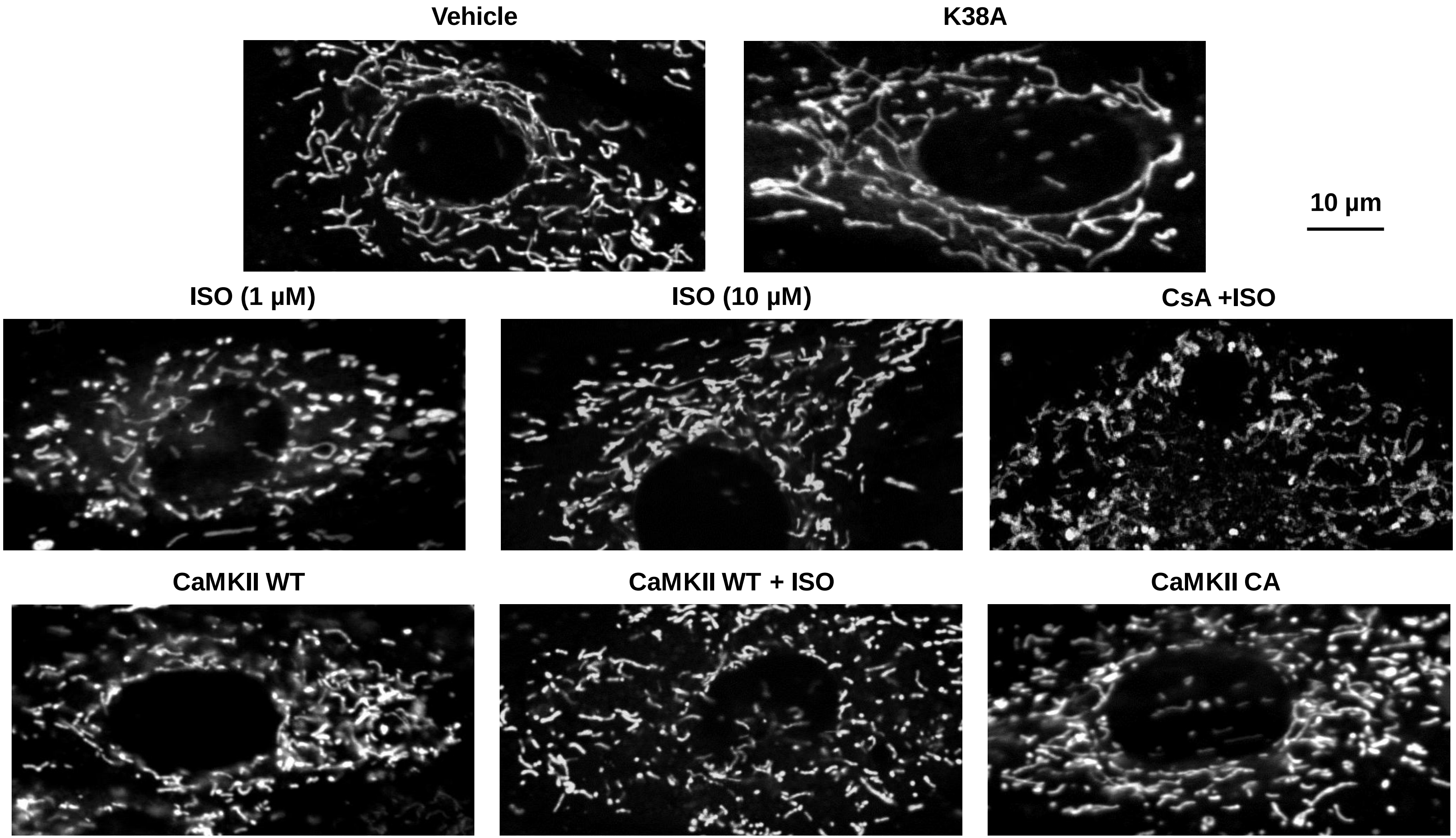


**Supplementary Figure 7.** Representative images showing the changes of mitochondrial morphology in H9C2 cells induced by various treatments. The mitochondrial morphology was monitored in live cells loaded with MitoTracker Green.

**Figure S7**

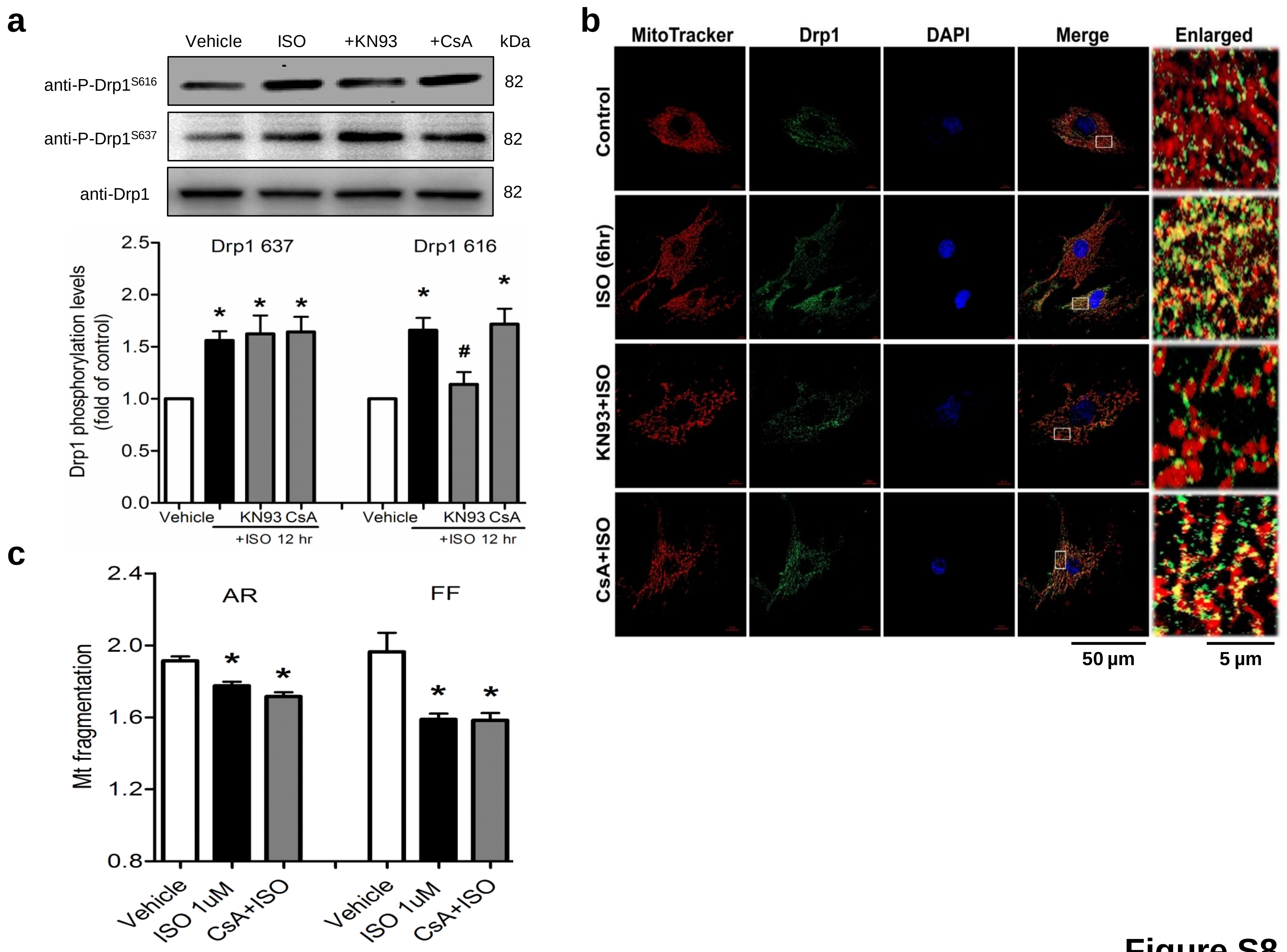

a
Vehicle
ISO
+KN93
+CsA
kDa
anti-P-Drp1S616
anti-P-Drp1S637
anti-Drp1
82
82
82
Drp1 637
Drp1 616
Drp1 phosphorylation levels (fold of control)
2.5
2.0
1.5
1.0
0.5
0.0
Vehicle
KN93 CsA
+ISO 12 hr
Vehicle
KN93 CsA
+ISO 12 hr
b
MitoTracker
Drp1
DAPI
Merge
Enlarged
Control
ISO (6hr)
KN93+ISO
CsA+ISO
50 μm
5 μm
c
AR
FF
Mt fragmentation
2.4
2.0
1.6
1.2
0.8
Vehicle
ISO 1uM
CsA+ISO
Vehicle
ISO 1uM
CsA+ISO


**Figure S8**

**Supplementary Figure 8.** CsA administration had no effects on ISO-induced Drp1 mitochondrial translocation and fragmentation in H9C2 cells. (**a**) CsA (1 µM) pretreatment had no effect on ISO (100 nM, 12 hr)-induced Drp1 phosphorylation at serine 616 and 637 sites in H9C2 cells. N = 3. (**b**) Representative immunofluorescent images showing CsA (1 µM) did not block ISO-induced Drp1 translocation to mitochondria in H9C2 cells. N = 3. (**c**) CsA (1 µM) also had no effect on ISO-induced mitochondrial fragmentation in H9C2 cells. N = 2246, 2695, and 1864 mitochondria in the groups of Vehicle, ISO and CsA+ISO, respectively. *P<0.05 versus Vehicle group, $^{\#}$P<0.05 versus ISO group. Data are mean $\pm$ s.e.m. The data were analyzed by using One-way ANOVA followed by Turkey post-test in (**a** and **c**).

**Figure S8**

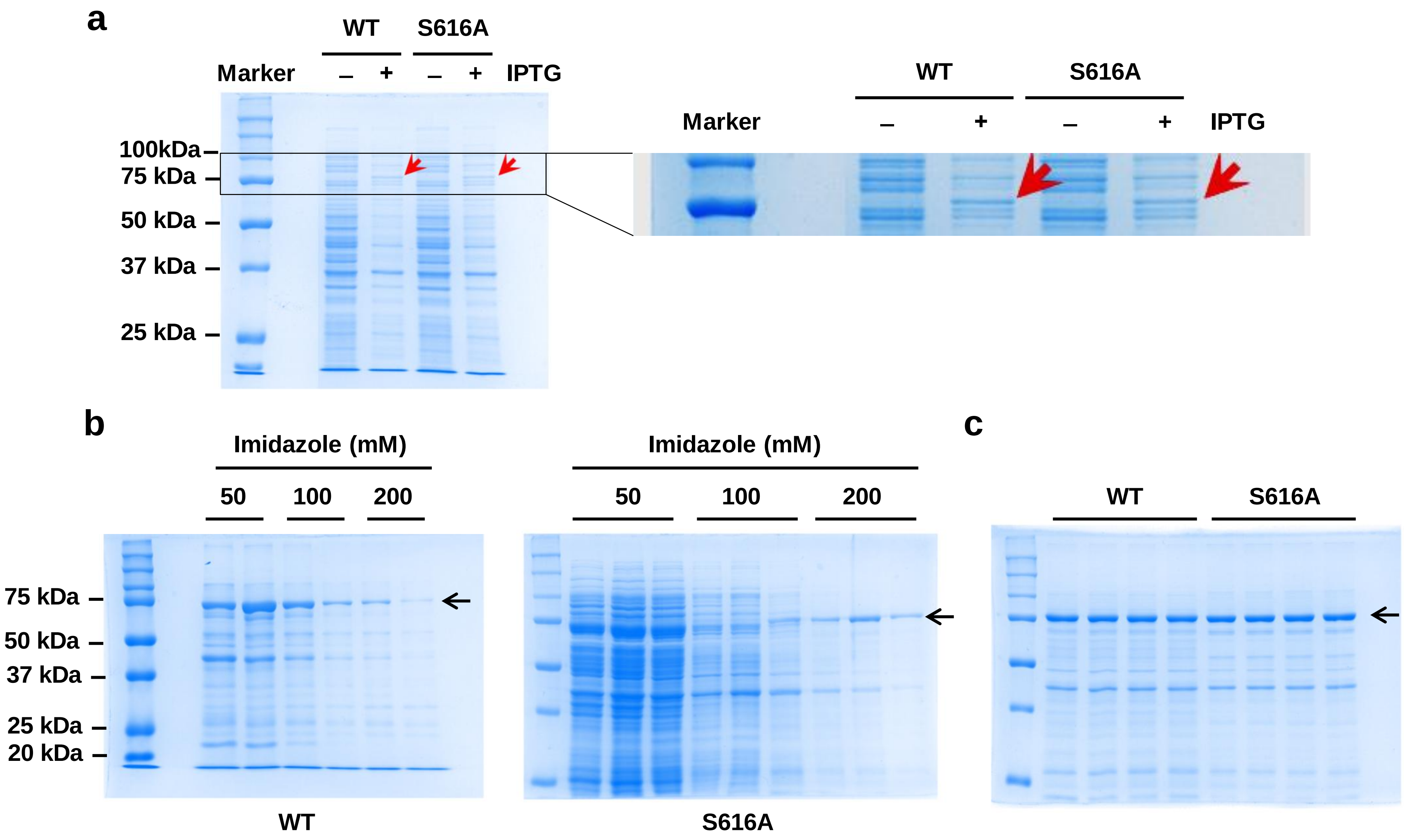
a
WT
S616A
Marker
–
+
–
+
IPTG
100kDa
75 kDa
50 kDa
37 kDa
25 kDa
WT
S616A
Marker
–
+
–
+
IPTG
b
Imidazole (mM)
50
100
200
75 kDa
50 kDa
37 kDa
25 kDa
20 kDa
WT
Imidazole (mM)
50
100
200
S616A
c
WT
S616A

# Figure S9

**Supplementary Figure 9.** Prokaryotic expression and purification of mouse wild type Drp1 and S616A mutation. (**a**) Crude cell lysates showing the induction of protein expression by IPTG. (**b**) Purification of wild type Drp1 and Drp1 S616A mutation. Imidazole was added after the crude lysates were incubated with Nickle resin to elute the proteins. The fraction after 200 mM imidazole were combined and used for *in vitro* phosphorylation assay. (**c**) Coomassie blue staining of the gel with the same loading of samples shown in Fig. 7**b**. Arrows indicate the corresponding Drp1 bands. Note, in the mouse sequence the mutation was done at S579 site, which is equivalent to S616 in human Drp1. For consistency and clarity we labeled the mutation according to human Drp1 sequence.

**Figure S9**

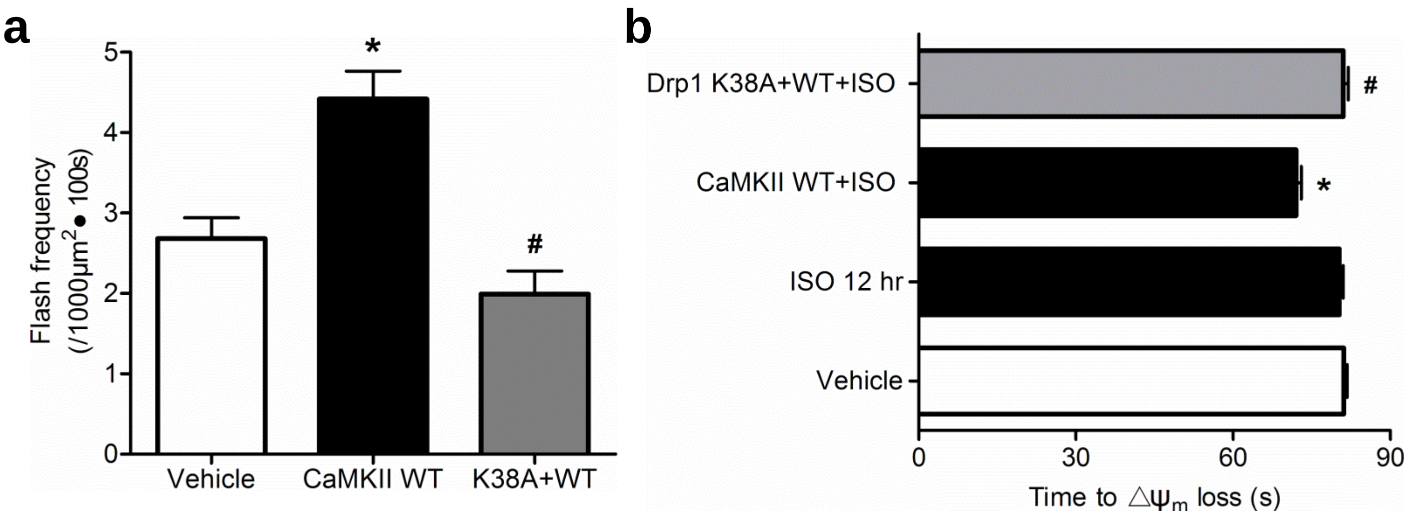


**Supplementary Figure 10.** Drp1 K38A prevented mPTP openings and mitochondrial stress induced by overexpression of CaMKII WT in adult cardiomyocytes. (**a**) Drp1 K38A blocked the increased flash frequency by overexpressing CaMKII WT. N = 14, 15, and16 cells in the groups of Vehicle, CaMKII WT and K38A+WT, respectively. (**b**) Drp1 K38A prolonged the time of laser-induced loss of $\triangle\psi_m$ in adult cardiomyocytes overexpressing CaMKII WT. N = 192-535 mitochondria from 15-48 cells. N = 535, 366, and 192 mitochondria from 15-48 cells in the groups of Vehicle, CaMKII WT and K38A+WT, respectively. Data in (**a, b**) are mean $\pm$ s.e.m. *P<0.05 versus Vehicle group, $^{\#}$P<0.05 versus CaMKII group. The data were analyzed by using One-way ANOVA followed by Turkey post-test in (**a-b**).

**Figure S10**

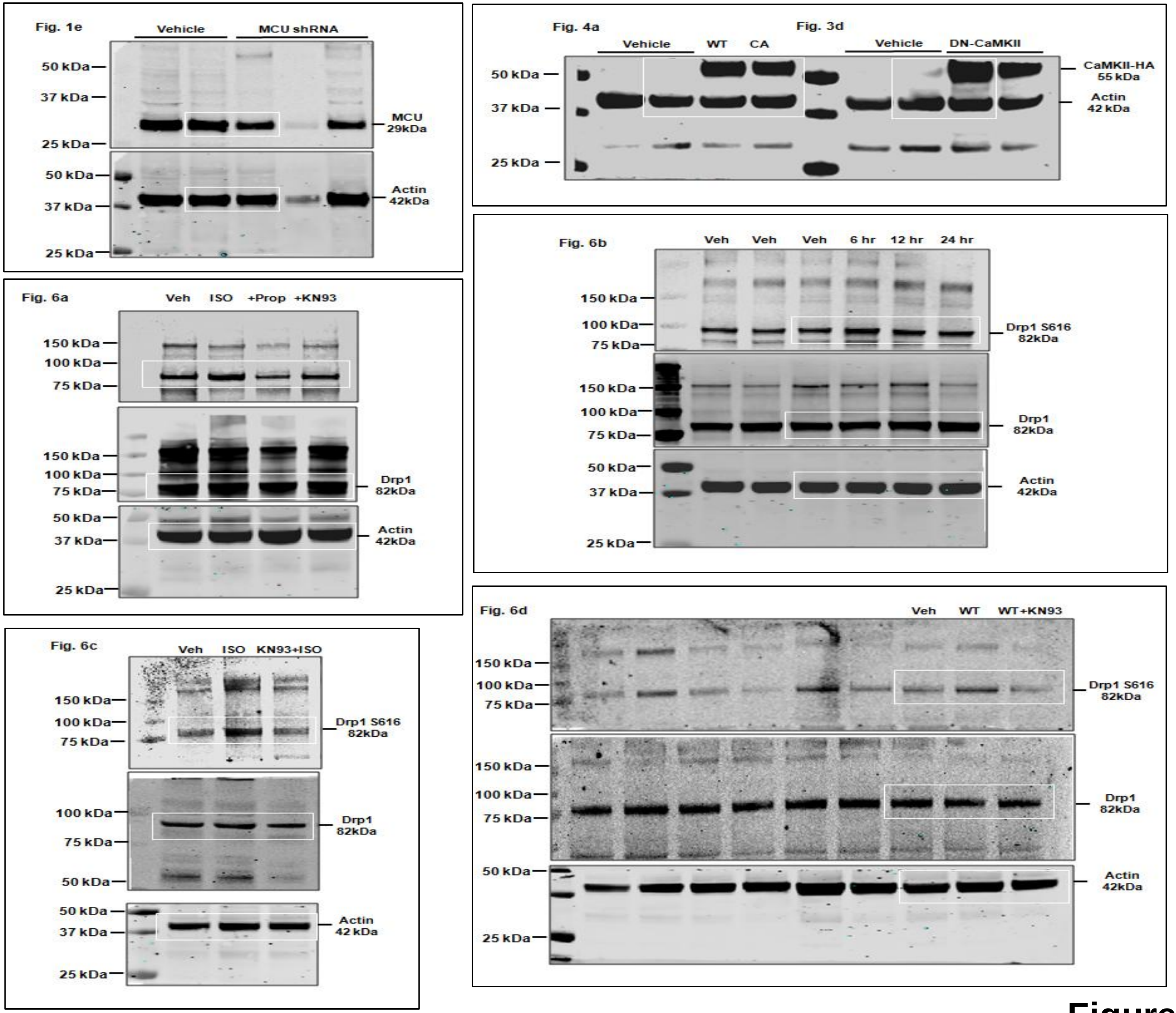
Fig. 1e
Vehicle
MCU shRNA
50 kDa
37 kDa
25 kDa
MCU 29kDa
50 kDa
37 kDa
25 kDa
Actin 42kDa
Fig. 4a
Vehicle
WT
CA
Fig. 3d
Vehicle
DN-CaMKII
50 kDa
37 kDa
25 kDa
CaMKII-HA 55 kDa
Actin 42 kDa
Fig. 6a
Veh
ISO
+Prop
+KN93
150 kDa
100 kDa
75 kDa
150 kDa
100 kDa
75 kDa
Drp1 82kDa
50 kDa
37 kDa
25 kDa
Actin 42kDa
Fig. 6b
Veh
Veh
Veh
6 hr
12 hr
24 hr
150 kDa
100 kDa
75 kDa
Drp1 S616 82kDa
150 kDa
100 kDa
75 kDa
Drp1 82kDa
50 kDa
37 kDa
25 kDa
Actin 42kDa
Fig. 6c
Veh
ISO
KN93+ISO
150 kDa
100 kDa
75 kDa
Drp1 S616 82kDa
100 kDa
75 kDa
50 kDa
Drp1 82kDa
50 kDa
37 kDa
25 kDa
Actin 42 kDa
Fig. 6d
Veh
WT
WT+KN93
150 kDa
100 kDa
75 kDa
Drp1 S616 82kDa
150 kDa
100 kDa
75 kDa
Drp1 82kDa
50 kDa
25 kDa
Actin 42kDa

**Figure S11**

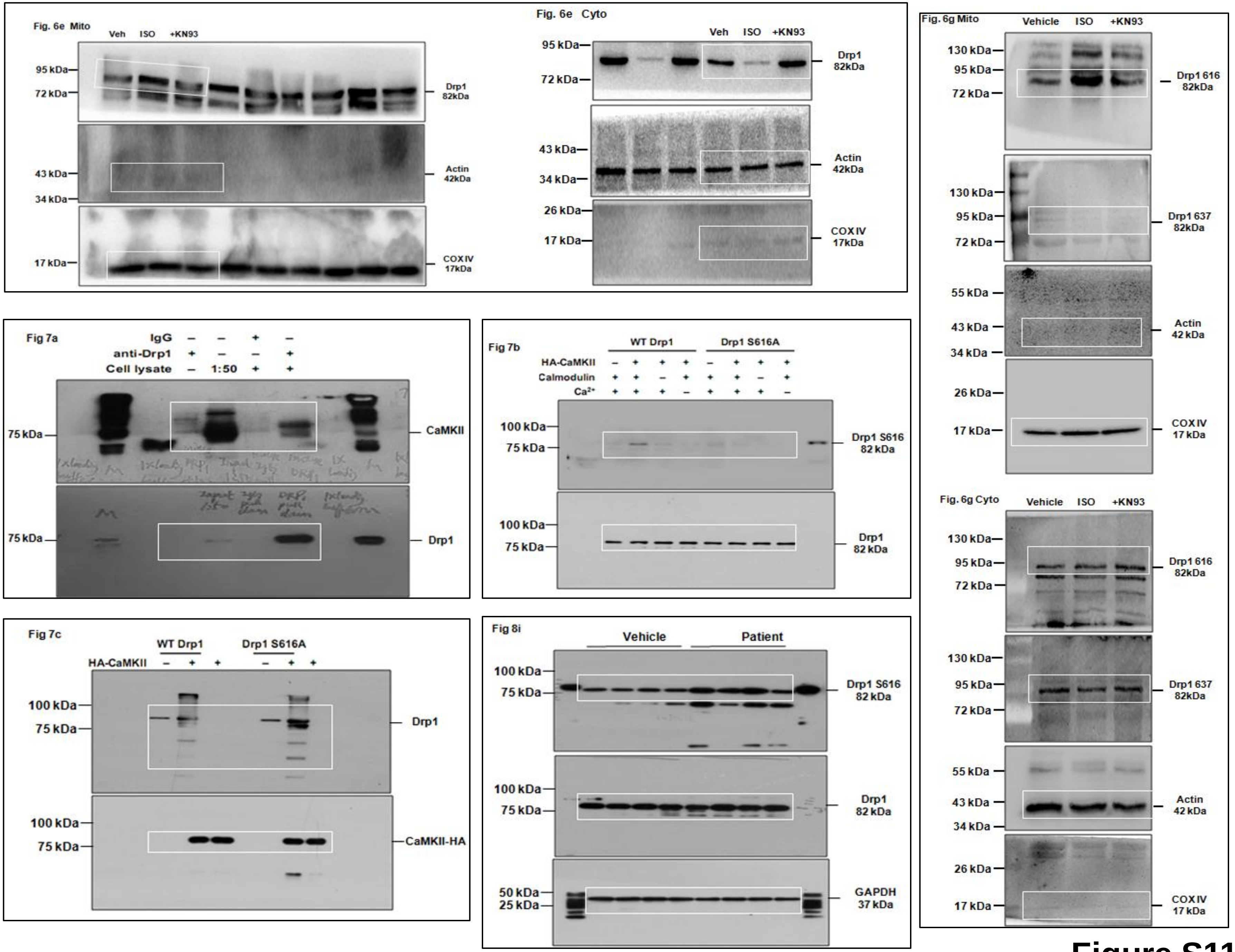
Fig. 6e Mito
Veh ISO +KN93
95 kDa
72 kDa
Drp1 82kDa
43 kDa
34 kDa
Actin 42kDa
17 kDa
COX IV 17kDa
Fig. 6e Cyto
Veh ISO +KN93
95 kDa
72 kDa
Drp1 82kDa
43 kDa
34 kDa
Actin 42kDa
26 kDa
17 kDa
COX IV 17kDa
Fig. 6g Mito
Vehicle ISO +KN93
130 kDa
95 kDa
72 kDa
Drp1 616 82kDa
130 kDa
95 kDa
72 kDa
Drp1 637 82kDa
55 kDa
43 kDa
34 kDa
Actin 42 kDa
26 kDa
17 kDa
COX IV 17 kDa
Fig. 6g Cyto
Vehicle ISO +KN93
130 kDa
95 kDa
72 kDa
Drp1 616 82kDa
130 kDa
95 kDa
72 kDa
Drp1 637 82kDa
55 kDa
43 kDa
34 kDa
Actin 42 kDa
26 kDa
17 kDa
COX IV 17 kDa
Fig 7a
IgG – – + –
anti-Drp1 + – – +
Cell lysate – 1:50 + +
75 kDa
CaMKII
75 kDa
Drp1
Fig 7b
WT Drp1
Drp1 S616A
HA-CaMKII – + + + – + + +
Calmodulin + + – + + + – +
Ca²⁺ + + + – + + + –
100 kDa
75 kDa
Drp1 S616 82 kDa
100 kDa
75 kDa
Drp1 82 kDa
Fig 7c
WT Drp1
Drp1 S616A
HA-CaMKII – + + – + +
100 kDa
75 kDa
Drp1
100 kDa
75 kDa
CaMKII-HA
Fig 8i
Vehicle
Patient
100 kDa
75 kDa
Drp1 S616 82 kDa
100 kDa
75 kDa
Drp1 82 kDa
50 kDa
25 kDa
GAPDH 37 kDa

**Figure S11**

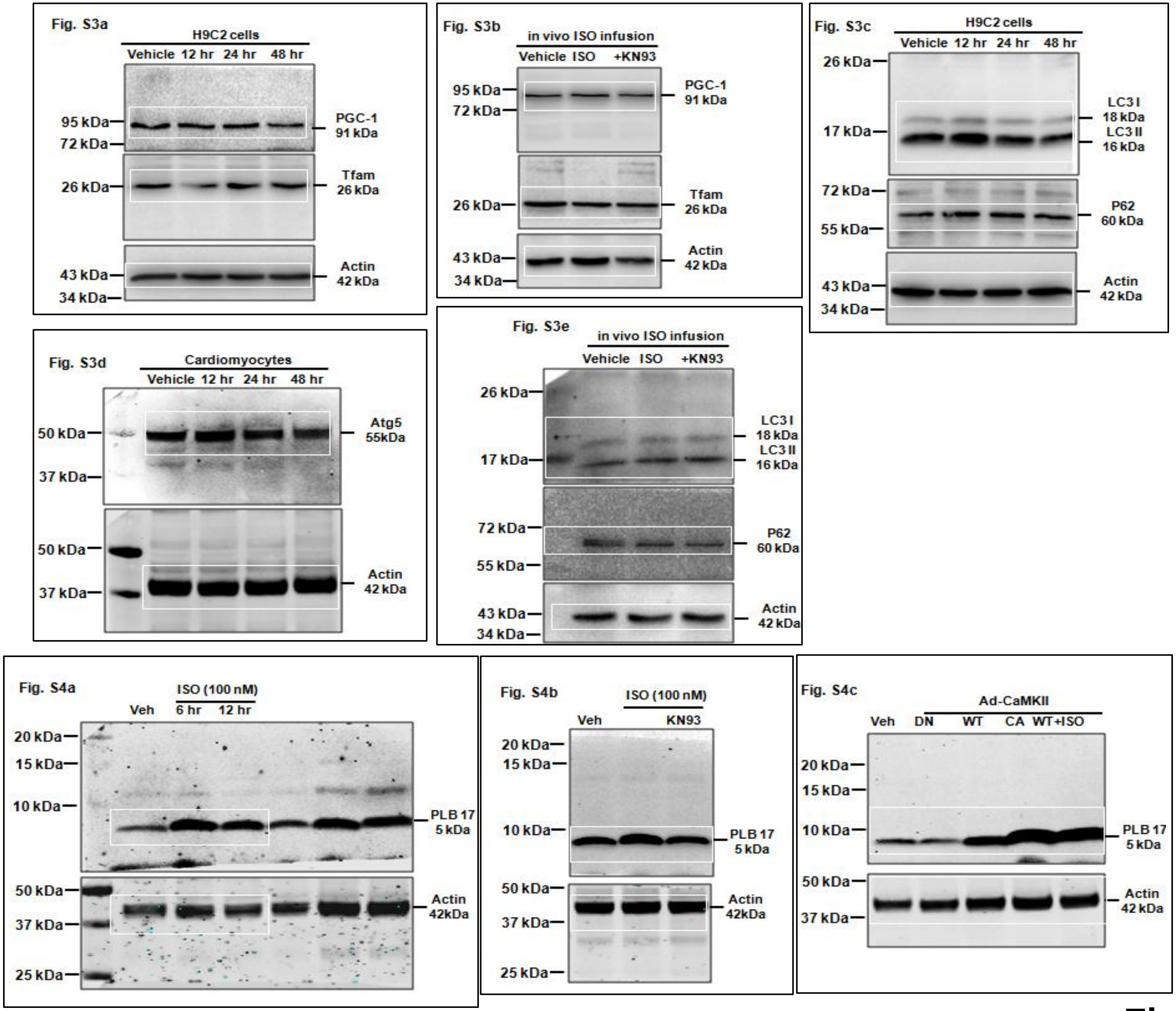
Fig. S3a
H9C2 cells
Vehicle 12 hr 24 hr 48 hr
95 kDa
72 kDa
PGC-1 91 kDa
26 kDa
Tfam 26 kDa
43 kDa
34 kDa
Actin 42 kDa
Fig. S3b
in vivo ISO infusion
Vehicle ISO +KN93
95 kDa
72 kDa
PGC-1 91 kDa
26 kDa
Tfam 26 kDa
43 kDa
34 kDa
Actin 42 kDa
Fig. S3c
H9C2 cells
Vehicle 12 hr 24 hr 48 hr
26 kDa
17 kDa
LC3 I 18 kDa
LC3 II 16 kDa
72 kDa
55 kDa
P62 60 kDa
43 kDa
34 kDa
Actin 42 kDa
Fig. S3d
Cardiomyocytes
Vehicle 12 hr 24 hr 48 hr
50 kDa
37 kDa
Atg5 55kDa
50 kDa
37 kDa
Actin 42 kDa
Fig. S3e
in vivo ISO infusion
Vehicle ISO +KN93
26 kDa
17 kDa
LC3 I 18 kDa
LC3 II 16 kDa
72 kDa
55 kDa
P62 60 kDa
43 kDa
34 kDa
Actin 42 kDa
Fig. S4a
ISO (100 nM)
Veh 6 hr 12 hr
20 kDa
15 kDa
10 kDa
PLB 17 5 kDa
50 kDa
37 kDa
25 kDa
Actin 42kDa
Fig. S4b
ISO (100 nM)
Veh KN93
20 kDa
15 kDa
10 kDa
PLB 17 5 kDa
50 kDa
37 kDa
25 kDa
Actin 42kDa
Fig. S4c
Ad-CaMKII
Veh DN WT CA WT+ISO
20 kDa
15 kDa
10 kDa
PLB 17 5 kDa
50 kDa
37 kDa
Actin 42 kDa

**Figure S11**

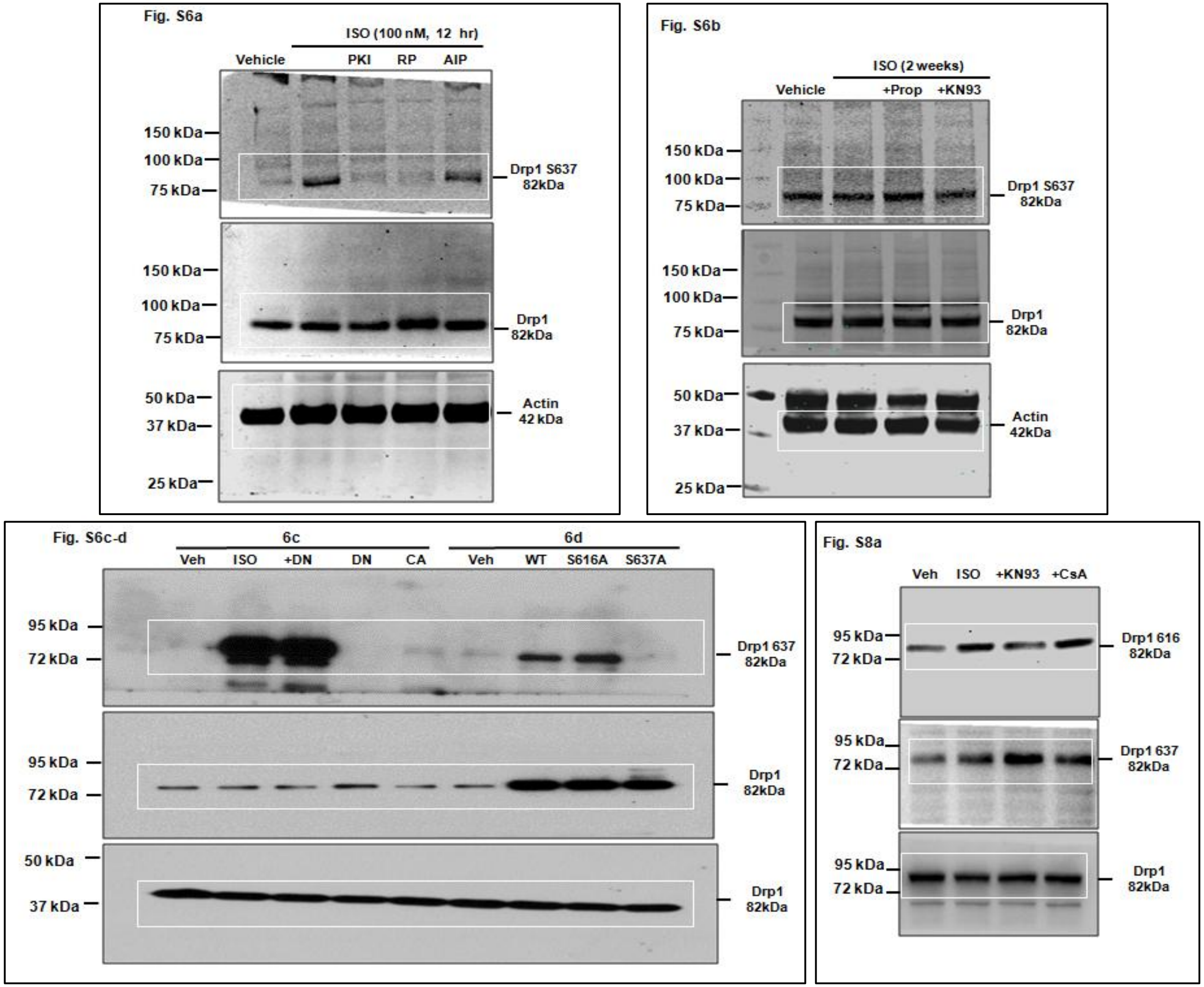


**Supplementary Figure 11.** Original un-cropped images of all the Western blots shown in the Figures and Supplementary Figures. The white boxes indicate the area/bands selected for the figures. We used protein ladders (Bio-Rad #161-0373 or Thermo Scientific #26617) to identify the molecular weight of the protein of interest.

**Figure S11**
